# The Potential for Extracellular Vesicles in Nanomedicine: A Review of Recent Advancements and Challenges Ahead

**DOI:** 10.1002/adbi.202400623

**Published:** 2024-12-31

**Authors:** Farbod Ebrahimi, Anjali Kumari, Samaneh Ghadami, Saqer Al Abdullah, Kristen Dellinger

**Affiliations:** ^1^ Department of Nanoengineering Joint School of Nanoscience and Nanoengineering North Carolina A&T State University 2907 E Gate City Blvd Greensboro NC 27401 USA

**Keywords:** biological nanoparticles, biomarkers, chronic diseases, diagnosis, exosomes, therapy

## Abstract

Extracellular vesicles (EVs) have emerged as promising tools in diagnostics and therapy for chronic diseases, including cancer and Alzheimer's. Small EVs, also called exosomes, are lipid‐bound particles (≈30–150 nm) that play a role in healthy and pathophysiological interactions, including intercellular communication, by transporting bioactive molecules, including proteins, lipids, and nucleic acids. Their ability to cross biological barriers, such as the blood‐brain barrier, makes them ideal candidates for targeted therapeutic interventions. In the context of chronic diseases, exosomes can be engineered to deliver active agents, including small molecules and siRNAs to specific target cells, providing a novel approach to precision medicine. Moreover, exosomes show great promise as repositories for diagnostic biomarkers. Their cargo can reflect the physiological and pathological status of the parent cells, making them valuable indicators of disease progression and response to treatment. This paper presents a comprehensive review of the application of exosomes in four chronic diseases: cancer, cardiovascular disease, neurodegenerative disease, and orthopedic disease, which significantly impact global public health due to their high prevalence and associated morbidity and mortality rates. Furthermore, the potential of exosomes as valuable tools for theranostics and disease management is highlighted. Finally, the challenges associated with exosomes and their demonstrated potential for advancing future nanomedicine applications are discussed.

## Graphical abstract

Extracellular vesicles (EVs) play a dual role in diagnostics and therapeutics, offering innovative solutions for treating cancer, cardiovascular, neurodegenerative, and orthopedic diseases. This review highlights EVs’ potential to revolutionize personalized medicine through specific applications in disease detection and treatment.

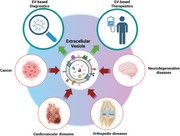

## 1 Introduction

In recent years, new strategies to enhance the efficacy of therapeutic agents and the reliability of diagnostic techniques have emerged through advances in nanomedicine, especially in chronic diseases.^[^ ^1^, ^2^ ^]^ Various synthetic nanomaterials, such as polymer‐based nanoparticles, liposomes, and noble metal nanoparticles, have been developed to deliver active agents for therapy and diagnosis efficiently. However, their use in clinical applications is limited due to toxicity, immunological clearance, and production problems. Natural nanoparticles, such as plant‐^[^ ^3^, ^4^ ^]^ and biofluid‐derived nanoparticles, could play an alternative role, which shows several advantages over synthetic nanoparticles regarding biocompatibility and targeted delivery.^[^ ^5^ ^]^ Exosomes, defined as cell‐released biological nanoparticles, are natural nanoparticles circulating as packaged cargo released from the cells and compartments that have the potential to overcome traditional limitations, such as rapid clearance, immunogenic issues, and toxicity. This makes exosomes a promising candidate for clinical administration.

**Figure 1:**
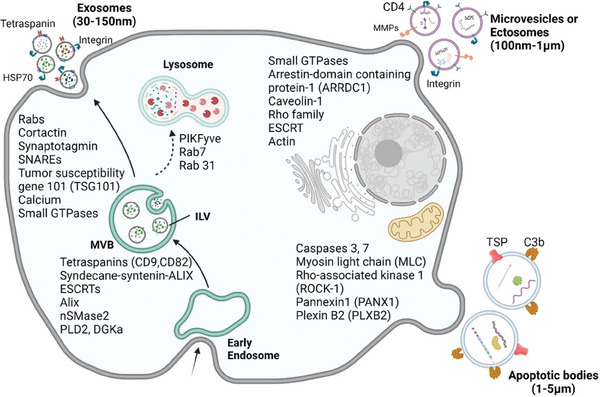
The different types of EVs, their markers and exosome biogenesis. A schematic illustration of the various types of EVs released by the cells including exosomes, ectosomes, and apoptotic bodies, into the extracellular environment. Exosomes are discharged from cells through a process called exocytosis, whereas ectosomes are released by cells through outward budding of the plasma membrane. Apoptotic bodies help clear cellular debris in late apoptosis. Exosome biogenesis begins with the invagination of the cell membrane to form the early endosome which then transforms into the late endosomes and finally forms the MVB containing intraluminal vesicles which mature into exosomes (created using Biorender.com).

Extracellular vesicles (EVs) are lipid bilayer‐enclosed membranous structures secreted by most eukaryotic cells under physiological and pathological conditions. They can carry lipids, proteins, receptors, and effector molecules to recipient cells.^[^ ^6^, ^7^ ^]^ EVs are typically categorized into three groups: apoptotic bodies (1–5 µm), microvesicles (MVs), also known as microparticles, ectosomes, or shedding vesicles (100 nm–1 µm), and exosomes (30–150 nm) (see **Table** **1** and **Figure** **1**).^[^ ^8^, ^9^, ^10^, ^11^ ^]^ Note that the field is moving away from the term exosomes and toward small EVs when the biogenesis mechanism is not known (which is the case in most studies); however, because most papers cited herein use the term “exosomes,” we will use that nomenclature for the most part herein.

**Table 1:** The key characteristics and differences of exosomes, microvesicles and apoptotic bodies.

	Exosomes	Microvesicles	Apoptotic bodies
Size (nm)	30–150	100–1000	1000–5000
Density (g/mL)	1.1–1.2	1.08–1.19	1.17–1.29
Origin	ILVs within MVBs	Plasma membrane and cellular content	Plasma membrane, cellular fragments
Morphology	Cup‐shaped, spherical, egg‐shaped, round	Heterogeneous	Heterogeneous
Contents	Proteins, mRNA, miRNA, lipids (sphingomyelin, Phosphatidylserine, cholesterol and ceramide)	Proteins, mRNA, miRNA, lipids (phosphatidylserine)	Cell organelles, proteins, nuclear fractions, DNA, coding/non‐coding RNAs, lipids, cytokines
Protein markers	Alix, TSG101, HSC70, heat shock (Hsp90, Hsp70, Hsp60), tetraspanins (CD9, CD63, CD81, CD82), CD37, LAMP1	Tetraspanins, cytoskeletal, CD40, heat shock, integrins, glycosylation and phosphorylation	Histones, HSP60, GRP78
Main functions	Cell‐to‐cell communication, neurodegeneration, coagulation, antigen presentation, diagnosis, and prognosis biomarkers	Cell‐to‐cell communication, diagnosis, and prognosis biomarkers	Assist to an efficient clearance of apoptotic cells, checking immune responses

Although this classification is based on size, biogenesis, cellular origin, content, and function, it can also vary widely.^[^ ^10^, ^12^, ^13^ ^]^ Apoptotic bodies are the largest and most heterogeneous population of EVs, which result after programmed cell death and can carry cell organelles, DNA, RNA, and proteins. Given different formation mechanisms, apoptotic bodies have been found to have extra protein profiles compared with exosomes and MVs.^[^ ^7^ ^]^ Because of the relatively large size of apoptotic bodies, they are not an ideal candidate to use as delivery systems. MVs are heterogeneous, membrane‐bound vesicles. MVs form from cells through the outward budding (blebbing) and fission from plasma membranes.^[^ ^14^ ^]^ The content of MVs is influenced by the parent cell, the surrounding environment, and the specific events or conditions prompting their release. In this context, “events or conditions” denote various internal or external stimuli, be they biochemical, physical, pathological, or related to cellular signaling, which leads to the release of MVs from the cell. They carry the membrane‐derived receptors, proteins, mRNA, miRNA, and lipids.^[^ ^8^ ^]^ Exosomes are the smallest vesicles and can be generated by most cells. Exosomes play a significant role in physiological and pathological processes. Generally, there are three stages of their formation: 1) the formation of endocytic vesicles from the plasma membrane, 2) the inward budding of the endosomal vesicle membrane resulting in multi‐vesicular bodies (MVBs) that contain intraluminal vesicles (ILVs), 3) MVBs are either degraded by lysosomes or fused with the plasma membrane, releasing the vesicular contents, known as exosomes.^[^ ^15^ ^]^ There are no specific protein markers to distinguish exosomes from MVs^7^. Despite the similar structures of exosomes and MVs, their size, shape, lipid composition, and cellular origin are distinct. The lipidomics of exosomes depend on cell origin; therefore, their lipid composition differs from MVs. Among EV subtypes, exosomes were selected as the primary drug delivery vehicle due to their unique physicochemical and biological properties. Exosomes enable superior tissue penetration, more consistent cellular internalization, and more predictable biodistribution.^[^ ^16^, ^17^ ^]^ Their smaller size minimizes the risk of vascular occlusion and facilitates more efficient cellular interactions.^[^ ^18^ ^]^ Additionally, exosomes offer remarkable molecular engineering potential, with a stable membrane composition that allows sophisticated surface modifications, high cargo loading efficiency, and reduced immunogenicity.^[^ ^19^ ^]^ These characteristics make exosomes particularly advantageous for targeted therapeutic applications, providing a more precise and controllable mechanism for drug delivery compared to both larger apoptotic bodies and MVs. Exosome membranes can contain glycosphingolipids, cholesterol, phosphatidylserine, and ceramide.^[^ ^13^, ^20^ ^]^ As Table 1 shows, common protein markers of exosomes, including Alix, TSG101, HSC70, heat shock (Hsp90, Hsp70, Hsp60), tetraspanins (CD9, CD63, CD81, CD82), CD37, and LAMP1.^[^ ^13^, ^21^ ^]^ The contents of intravesicular exosomes and membranes vary depending on their host cell origin, potentially serving as biomarker repositories for diagnosis and therapy monitoring.^[^ ^21^ ^]^ Due to their structure and function, they have also been proposed to be used as nanocarriers for delivering active agents.^[^ ^13^ ^]^

To effectively use exosomes in biomedical applications, it is important to consider the methods of isolation, purification, and characterization. The most common isolation techniques include multiple‐step centrifugation, density gradient ultracentrifugation, size exclusion chromatography, ultrafiltration, tangential flow filtration, magnetic separation, mass spectrometric immunoassay, and polymer‐based precipitation.^[^ ^22^, ^23^ ^]^ Purity, cost‐effectiveness, throughput, and efficiency are the most critical features of isolation. There are also various techniques available for the analysis of exosomes. Methods to determine their molecular content include western blot, ELISA, RT‐qPCR, and next‐generation sequencing. Nanoparticle tracking analysis and resistive pulse sensing can be used to analyze their size distribution and concentrations. Through labeling of exosomes with specific antibodies and fluorescent reporters, the type and amount of protein expression can be evaluated by nano‐flow cytometry. Imaging and characterization techniques comprise dynamic light scattering, transmission electron microscopy, scanning electron microscopy, and atomic force microscopy that can be used to visualize the overall structure of exosomes, including size and morphology.^[^ ^7^, ^21^ ^]^

In this review, we present a comprehensive discussion and highlight the state‐of‐the‐art in exosome‐based applications in nanomedicine. We focus on four categories of health‐related issues: cancer, cardiovascular disease (CVD), orthopedic disease (OD), and neurological disease (ND). Finally, we discuss recent advances in detecting exosomes using biosensing platforms based on nanomaterials, as well as the challenges and prospects in the field.

Exosomes are involved in physiological conditions, such as cellular communication,^[^ ^6^ ^]^ blood coagulation,^[^ ^10^ ^]^ antigen presentation to T cells,^[^ ^24^ ^]^ and stimulation of the immune response.^[^ ^22^ ^]^ In pathological conditions, they play a significant role in different diseases, including cancer,^[^ ^22^, ^25^ ^]^ CVD,^[^ ^26^ ^]^ ND,^[^ ^6^, ^27^ ^]^ and inflammation.^[^ ^28^ ^]^ For example, exosomes secreted by pancreatic cancer cells can reprogram cells in the tumor microenvironment, which leads to an increase in pancreatic cancer cell proliferation and migration.^[^ ^25^ ^]^ In another example, cancer‐associated fibroblasts, observed within the tumor microenvironment, secrete exosomes. They can affect the proliferation, migration, and invasion of cancer cells, which leads to cancer progression.^29^ These findings highlight the role of exosomes in tumor growth, which might lead to the discovery of novel active agents to suppress the secretion of exosomes from cancer cells. In addition, combining exosomes with immunotherapy could enhance anti‐tumor immunity, inhibiting tumor development.^[^ ^30^ ^]^ Furthermore, exosomes show promise for diagnosing and treating CVD and OD. Contents of exosomes, such as different RNA types, might be used as diagnostic biomarkers or even therapeutic agents for CVD and OD.^[^ ^11^, ^26^, ^31^ ^]^ Recent studies have also demonstrated that exosomes have diverse functions in ND pathology^[^ ^32^, ^33^ ^]^ and may have significant potential as diagnostic ND biomarkers.^[^ ^2^, ^32^, ^34^ ^]^ Exosomes may also be used as therapeutic agents to deliver cargo to cells and could be modified to serve as a drug delivery system to treat NDs.^[^ ^35^, ^36^ ^]^ Given that exosomes have unique properties such as their natural origin, high biocompatibility, and small size, they can be utilized as drug delivery systems and labeling nanoprobes for cellular and molecular imaging, disease monitoring, and pharmacokinetic tracking.^[^ ^37^, ^38^, ^39^ ^]^

**Figure 2:**
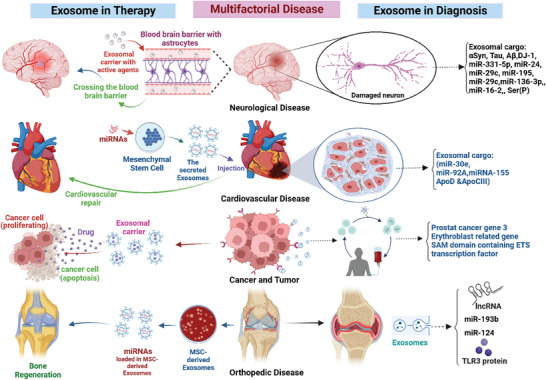
Schematic of diverse functions of exosomes in theranostics of chronic diseases (created using Biorender.com).

In the next section, we describe these diverse functions of exosomes in nanomedicine by focusing on drug delivery and diagnosis in four major categories of chronic diseases (**Figure** **2**).

## 2 Exosome‐Mediated Therapeutic Strategies for Chronic Diseases

**Figure 3:**
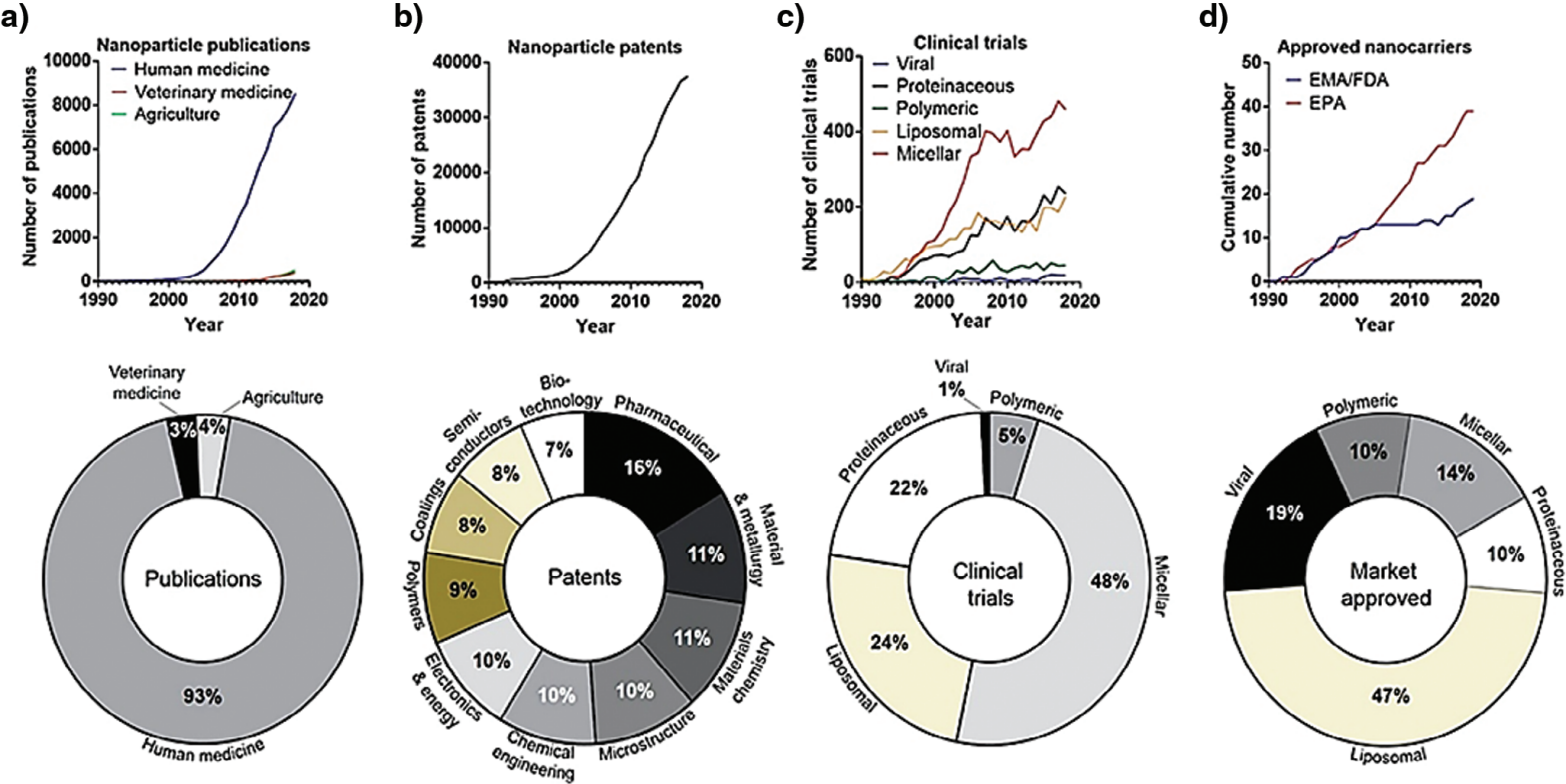
The timeline and corresponding distribution of research papers, patents, and clinical applications related to the utilization of synthetic nanomaterials for drug delivery. Reproduced with permission,^[^ ^40^ ^]^ 2020 American Chemical Society.

In the last decade, several synthetic nano‐drug delivery systems have been developed to enhance the efficacy of therapeutic agents and reduce side effects. The majority of authorized synthetic nanocarriers are based on liposomes (47%), followed by micellar (14%) and polymeric (10%) formulations.^[^ ^40^ ^]^ Noble metal nanoparticles, dendrimers, fullerene, nanocapsules, solid‐lipid nanoparticles, and carbon nanotubes are among the other particularly noticeable examples of synthetic nano‐delivery systems.^[^ ^41^, ^42^, ^43^, ^44^ ^]^ However, since 1990, only 21 formulations of nanocarriers have made it to the market and received approval from the US Food and Drug Administration (FDA) and the European Medicines Agency for medical use (**Figure** **3**).^[^ ^40^ ^]^ Currently, synthetic nanocarriers have significant limitations, including low bioavailability of active agents in aqueous solutions, the degradation of active agents in vivo, and inappropriate dosage delivery of active agents in target sites, factors that impact the effectiveness of the treatment. Moreover, serum proteins commonly adsorb onto the surface of synthetic nanocarriers after they enter the bloodstream, thus forming the protein corona, which is a great challenge for the efficacy of artificial drug delivery systems.^[^ ^12^ ^]^ These challenges lead to inefficiency, immunological clearance, off‐target effects, and cytotoxicity.^[^ ^12^, ^36^ ^]^

Endogenous nanocarriers, especially exosomes, could play an alternative role in drug delivery. The common features of exosomes with synthetic nanoparticles include nano‐sized dimension, drug loading capacity, modifiability, and functionalization capability, and carrying different active and targeted delivery agents. However, exosomes have specific features that distinguish them from synthetic nanocarriers by offering considerable advantages over their synthetic counterparts, including low immunogenicity, proper tissue penetration, long circulation half‐life, and crossing the biological protection system such as blood‐brain barrier (BBB).^[^ ^12^, ^36^, ^45^, ^46^, ^47^ ^]^ They are potentially less toxic than synthetic nanoparticles.^[^ ^48^ ^]^ Compared with synthetic nanocarriers, exosomes originate from the body and have better biocompatibility and lower immunogenicity. They can be broadly distributed and stabilized in the biofluids due to the expression of CD55 and CD59 on their surface. This prevents the activation of opsonin and coagulation factors and may avoid rapid clearance from circulation, thereby increasing their bioavailability.^[^ ^49^, ^50^ ^]^

Additionally, exosomes maintain the architecture of parent cells’ membrane proteins, which retain progenitors’ intrinsic targeting properties. Because of their communicative ability, exosomes can enter the targeted cell using endocytosis, phagocytosis, macropinocytosis, lipid raft‐mediated internalization, and fusion, which leads to the release of loaded active agents into the cytoplasm.^[^ ^51^, ^52^ ^]^ The various proteins on the surface of exosomes provide heterogeneous structures, enabling them to penetrate the cells in various ways after interaction with cells.^[^ ^49^ ^]^ It is a great challenge for synthetic nanoparticles to pass through the cell wall.^[^ ^37^ ^]^ In fact, exosomes deliver their cargo to the target cell by avoiding immunological activation or silencing an immune response from the host.^[^ ^53^ ^]^ In most cases, the binding of exosomes to the recipient cells is achieved by specific interactions between proteins on the surface of exosomes and receptors on the plasma membrane of the recipient cells.^[^ ^54^ ^]^ Thus, the source of exosomes must be selected individually based on the target disease. Another advantage of exosomes as a drug delivery system could be their weak nonspecific interactions with circulating proteins compared with synthetic nanocarriers.^[^ ^12^ ^]^

In drug loading, active agents can be loaded into exosomes through pre‐secretory or post‐secretory approaches. Pre‐secretory active agent loading or in vivo loading refers to the loading of the active agents onto the parent cell before it secretes modified exosomes. This method is preferred for loading high molecular weight proteins and RNAs such as mRNA.^[^ ^53^ ^]^ The main drawbacks of this approach can be described as follows: 1) it is not yet possible to regulate the loading efficiency of active agents, and 2) the function of exosomal membrane proteins might be impaired.^[^ ^49^, ^53^ ^]^ Post‐secretory or in vitro loading is the process of adding active agents directly to exosomes in a specific manner after exosome isolation. This approach is more efficient than in vivo loading, giving more control over the encapsulation effectiveness of the final cargo and loading capacity.^[^ ^53^ ^]^ However, exosome aggregation, membrane degradation, and limited yield are among the disadvantages of in vitro loading.^[^ ^49^ ^]^ Gene editing and progenitor cells belong to the pre‐secondary way. Electroporation, sonication, extrusion, saponin‐assisted treatment, freeze and thaw cycles, and co‐incubation with exosomes are all common techniques used to load various active agents in the post‐secondary approach.^[^ ^49^ ^]^ The selection of an appropriate loading method is often based on the exosome origin regarding lipid composition and membrane structure, efficiency, and active agent properties, such as its relative hydrophilicity/hydrophobicity.^[^ ^37^, ^49^ ^]^

Exosomes have been derived from different types of parental cells, such as dendritic cells (DC), mesenchymal stem cells (MSC), macrophages, red blood cells, B lymphocytes, monocytes, neurons, intestinal epithelial cells, etc.^[^ ^22^ ^]^ Like liposomes, the exosome structure comprises a natural bilayer membrane with an aqueous core and highly depends on their origin. This means that hydrophilic and lipophilic drugs can be loaded into the core and phospholipid bilayer membranes, respectively (**Table** **2**). However, due to the low total area of the membrane that prevents a high drug encapsulation, it is not suitable for high drug dosage in the membrane. Additionally, during a long‐term course of treatment, the drug release from the membrane cannot be regulated in the body. Tran PHL et al. demonstrate a new strategy in vitro for transforming aspirin, a poorly water‐soluble drug, through preferably nano‐amorphous structure composed of both a hydrophilic and a hydrophobic moiety that can be embedded in both hydrophilic and hydrophobic sections of the exosomes to overcome the challenge of limited encapsulation efficiency of exosomes’ bilayer.^[^ ^55^ ^]^

**Table 2:** Summary of the variety of exosome‐based drug delivery systems.

Cargo	Therapeutic agent	Method(s) of drug loading	Exosome origin	Route of administration	Function	Ref
Proexosomes	Aspirin (lipophilic)	Incubation	HT29	In vitro	anticancer	[55]
Small molecule	miRNA	electroporation	HEK293 cells	injection	malignant neoplasms	[56]
RNA	RNA	–	MSCs	intra‐articular injection	cartilage repair	[57]
Small molecule	Calcein (lipophilic)	Incubation	HeLa cell	in vitro	anticancer	[58]
Protein	catalase	incubation, freeze‐thaw, sonication, extrusion	mouse macrophage cell line (Raw 264.7)	intranasal	PD treatment	[36]
Protein	MSC exosomes	molecular cloning	MSCs	intraperitoneal injection	Repair after MI	[59]
Protein	BACE1 siRNA	electroporation	DCs	injection	AD treatment	[60]
Small molecule	DOX (hydrophilic)	electroporation	DCs of mouse	intravenously	Tumor targeting	[47]
Small molecule	PTX (lipophilic), DOX (hydrophilic)	sonication	mouse macrophage cell line (Raw 264.7)	injection	MDR cancer	[61]
RNA	miRNAs	surface engineering	medicinal plant *(Pueraria lobate)*	intranasal	PD treatment	[62]
Small molecule	flurbiprofen	surface engineering	MSCs	in vitro	Bone implants	[63]
Protein	cleaving enzyme 1 (*Bace1*) and Cre recombinase	photocleavage	Neuronal exosomes	intravenously	AD treatment	[64]
RNA	miRNA‐146a	electroporation	Milk‐derived exosomes	intravenously	MI‐reperfusion injury	[65]

Drug delivery using exosomes is very adaptable and compatible. Various administration routes are available for exosomal drug delivery, including intravenous injection,^[^ ^47^ ^]^ subcutaneous injection, intraperitoneal injection,^[^ ^59^ ^]^ intraarticular injection,^[^ ^57^ ^]^ inhalable and nasal administration,^[^ ^5^, ^36^ ^]^ and oral administration.^[^ ^66^ ^]^ The selection of administration methods depends on the targeted site, clearance rate, active agents’ pharmacokinetics and pharmacodynamic profile, and convenience and compliance. Advances in active agent delivery with exosomes show many opportunities to develop new methods to treat different human diseases.

The targeting specificity is mainly attributed to the surface characteristics of exosomes. The surface modification and engineering of exosomes can impart additional functionalities such as targeted delivery, immunological reagents, encapsulation, or conjugation of active agents.^[^ ^62^, ^64^, ^67^, ^68^ ^]^ There are two approaches for modifying exosomes: direct chemical and indirect modifications through biological/genetic means. The chemical modification of exosomes depends on the biological binding of targeted ligands to surface proteins, which could lead to surface protein inactivation or exosome aggregation. It is possible to covalently link targeting molecules to the exosomal surface or use non‐covalent techniques.^[^ ^69^ ^]^ Targeting peptides and polyethylene glycol (PEG) are among the common chemical modifications of exosomes.^[^ ^69^, ^70^ ^]^ Exosome biological/genetic modification is an excellent strategy for displaying functional ligands on the exosome membrane. The modification of exosomes can be achieved indirectly by stimulating the parent cells to produce genetically modified exosomes. However, it requires plasmid construction and overexpression of proteins in parent cells.^[^ ^68^ ^]^ Table 2 summarizes typical examples of exosome‐based drug delivery systems for various chronic diseases. Promising results from these systems indicate great potential for them to be endogenous and versatile nanocarriers for the advanced delivery of various drugs.

In fact, the origin of exosomes, the loading method of active agents, administration route, and surface engineering of exosomes are the primary factors that should be particularly taken into consideration to increase the efficacy, safety, and specific site‐targeting of exosome‐based drug delivery to obtain optimal therapeutic impact. The function of exosomes is highly dependent on the donor cells. Therefore, the appropriate selection of exosomes as nanocarriers can significantly enhance targeted drug delivery. The loading capacity of active agents can be improved by selecting a proper loading method. The delivery of sufficient dosage of active agents can be enhanced by picking the correct administration route. Additionally, surface‐modified exosomes can simultaneously improve loading efficiency and specific site targeting and prevent damage to normal tissue and cells.^[^ ^67^ ^]^ Therefore, combining the multifactorial advantages of exosome‐based drug delivery can achieve the best therapeutic effect.

### 2.1 Active Agents Delivered by Exosomes in Chronic Diseases

Exosomes are capable of delivering different cargoes, including nucleic acids (mRNA, miRNA, siRNA, DNA), peptides and proteins (enzymes, cytoskeletal proteins, transmembrane proteins), lipids, and small molecules (curcumin, antibiotics, paclitaxel (PTX), doxorubicin (DOX), calcein).^[^ ^46^, ^56^, ^57^, ^58^, ^71^ ^]^ Different types of hydrophobic and hydrophilic active agent molecules can be encapsulated into the exosomes or conjugated on the surface of exosomes and delivered to the site of interest (Table 2).

Exosomes can carry small molecules as active agents, which could increase their effectiveness by enhancing cellular uptake and recognition. They improve the traditional delivery‐related problems of small molecule active agents regarding low therapeutic index and poor bioavailability.^[^ ^72^ ^]^ For example, a couple of powerful anticancer active agents, particularly PTX, DOX, and curcumin, which can inhibit and suppress the proliferation of several types of cancer cells, have been delivered by means of exosomes with high efficacy.^[^ ^61^, ^72^ ^]^ Through targeted delivery, exosomes could prevent the cytotoxic side effects of small molecular weight active agents and damage to healthy cells.

Nucleic acids can be used as active agents for gene therapy and vaccines, which are rapidly developed in medicine, leading to adjustments in their initial steps toward clinical application. Moreover, RNA‐based therapies could be applied to chronic disorders and viral infections, such as the most recent RNA‐based COVID‐19 vaccines. However, the integrated delivery of RNAs, especially miRNA and siRNA, is generally ineffective because of the fast degradation and clearance of these nucleic acids and often triggers an innate immune response, which depends on their sequence. Moreover, miRNA and siRNA are hydrophilic, which means they cannot cross the hydrophobic cell membrane to the cytoplasm.^[^ ^40^ ^]^ These problems can be eliminated by encapsulating these nucleic acids in exosomes. Exosomes are a promising candidate for the delivery of different types of nucleic acids due to their ability to protect their cargo from degradation in an extracellular environment and deliver it to recipient cells, as well as the possibility of surface modification in terms of targeting delivery.^[^ ^73^ ^]^

Proteins and peptides have been used for surface modification of exosomes or as active agents, especially in NDs, for delivery of neuroprotective proteins such as nerve growth factors and neurotrophic factors.^[^ ^40^ ^]^ The ability of exosomes to cross the BBB makes them a promising cargo to deliver these proteins to the central nervous system. Furthermore, the surface‐engineered exosomes with specific peptides and proteins could enhance the conjugation of active agents with exosome membranes. Surface‐modified exosomes with peptides can also act as targeting ligands to direct exosomes to disease sites, which is particularly beneficial for cancer treatment and immunotherapy.^[^ ^67^ ^]^ Moreover, the targeted delivery of active agents into the brain can be achieved through surface‐engineered exosomes containing targeting peptides.^[^ ^70^ ^]^

Considering that exosomes can carry different types of active agents, they are applied to the treatment of numerous disorders and injuries such as chronic respiratory diseases,^[^ ^71^, ^74^ ^]^ diabetics, kidney injury,^[^ ^11^ ^]^ hepatitis,^[^ ^75^ ^]^ etc. For instance, a recent in vivo experiment demonstrates that taking advantage of engineered miR‐31 exosomes promotes the healing of diabetic wounds by enhancing angiogenesis, fibrogenesis, and reepithelization.^[^ ^76^ ^]^ However, in order to have accurate and better comprehension in this review, the main functions of exosomes in therapeutic approach have been categorized into four major fields: 1) Cancer and tumor therapy,^[^ ^47^, ^51^, ^56^ ^]^ 2) CVD,^[^ ^26^, ^59^ ^]^ 3) OD,^[^ ^57^ ^]^ and 4. NDs such as Alzheimer's disease (AD) and Parkinson's disease (PD).^[^ ^36^, ^60^ ^]^ Figure 2 illustrates the ability of exosomes to enhance each field's therapeutic goal.

#### 2.1.1 Cancer

Cancer is among the most fatal diseases worldwide. with a high mortality and incurability rate. In 2021, nearly 10 million individuals died from cancer and 19.29 million new cases were identified.^[^ ^68^ ^]^ Radiation therapy, chemotherapy, targeted therapy, immunotherapy, and surgery are currently available clinical cancer treatment methods, and among them, chemotherapy and radiation therapy are the most widely applied clinical approaches, for preventing DNA synthesis and mitosis, which results in the death of cancer cells that are actively growing and dividing.^[^ ^30^, ^52^ ^]^ Despite their undisputed contribution, the drawbacks of these invasive and non‐cancer cell‐selective approaches, which cause severe undesired side effects, include adverse reactions, high recurrence rates, damage to healthy tissues, drug resistance, and long‐term difficulties. For example, because of the poor tissue penetration of chemotherapeutic drugs, higher doses are required, which leads to elevated toxicity in normal cells, increases the adverse effect and deteriorates the therapeutic efficacies.^[^ ^52^ ^]^ Consequently, the necessity for efficient and safe cancer treatment strategies is paramount.^[^ ^68^ ^]^

To improve therapeutic efficacies, various types of nano‐drug delivery systems have been introduced and numerous studies on targeted therapy and nanotechnology have been conducted with respect to targeting cancer treatment over the past 10+ years. Synthetic nanocarriers have played a major role in targeted therapies for cancer and a few nanocarrier formulations were authorized by the FDA for clinical applications. High cost, low specificity, toxicity, elimination by the immune system and degradation in the bloodstream are some of the limitations that narrowed the wide application of synthetic nanocarriers to cancer treatment. Recently, exosomes have been significantly highlighted as a novel drug delivery system for cancer therapy. The tissue selectivity, safety, crossing biological barriers and stability of exosomes make them advantageous as drug delivery systems. They are able to deliver active agents through the target cells’ plasma membranes and into the correct cellular compartment to exert a functional response.^[^ ^52^ ^]^ They have been employed to treat in vitro and in vivo studies for different types of cancer such as lung, breast, colon, prostate, etc. As mentioned in the previous section, various types of anti‐cancer active agents could be delivered by exosomes, e.g., nucleic acids, small molecules and proteins, which demonstrate a promising therapeutic outcome in animal models. Because of the low toxicity and immunogenicity of exosomes, they can serve as nanocarriers for cytotoxic anti‐cancer active agents, such as PTX, DOX and docetaxel as well as other chemotherapeutic active agents, e.g. celasterol and curcumin, with more stability and higher specificity for targeted tumor cells.^[^ ^30^, ^47^, ^61^, ^77^ ^]^

They could be used as activators of adaptive immune response in the tumor environment, leading to enhanced anti‐tumor response in cancer immunotherapy. They are able to stimulate antitumor immune response and modulate tumor cell proliferation.^[^ ^78^ ^]^ The biologically active cargoes on exosomes, such as MHC and costimulatory molecules, have been shown to participate in exosome‐mediated anti‐cancer immune responses and could be employed as immunotherapeutic vaccines.^[^ ^30^ ^]^ Exosomes derived from natural killer immune cells have a significant anti‐tumor effect. It has been reported that CD8^+^ T cell‐derived exosomes could activate the innate immune response which is beneficial for tumor immunotherapy.^[^ ^79^ ^]^ In a recent study, the surface‐engineered exosomes with CD62L (a gene for lymphocyte homing to lymph nodes) and OX40L (a gene for regulation of T cells) could activate effector T cells, consequently amplifying the antitumor immune response and inhibiting tumor development.^[^ ^80^ ^]^ In addition, interest in exosome‐based anticancer vaccines has increased. Exosomes can be utilized as active specific immunotherapy due to the recent clinical validation and approval of various antibodies that neutralize immune checkpoint receptors.^[^ ^78^, ^81^, ^82^ ^]^

Exosomes have a great potential for RNA‐based cancer treatment/gene therapy targeting the cancer cells and inhibition of tumor growth. It has been reported that exosomes successfully delivered functional mRNAs to treat breast cancer in vitro and leukemia in vivo without any toxicity, which could suppress tumor growth and expression of cancer‐related miRNAs, respectively.^[^ ^83^, ^84^ ^]^ Further, exosomes mediate miRNA delivery which involves controlling the expression of cancer‐related genes by the miRNA complementation, which serves through two mechanisms, 1) by introducing exogenous miRNAs known to promote tumor inhibition and migration of cancer cells as miRNA replacement or 2) by providing specific miRNA inhibitors to suppress tumor promoting miRNA as miRNA inhibition.^[^ ^68^ ^]^ Exosomes mediate the delivery of therapeutic siRNA to block the expression of key genes related to cancer cell proliferation, drug resistance, immune checkpoints and tumor microenvironment, which leads to tumor suppression. The combination of RNA‐based exosomal nanocarrier with chemotherapy and immunotherapy provides a synergistic outcome, leading to reduced side effects and improved quality of life.^[^ ^68^, ^79^ ^]^

To enhance tumor targeting and tissue‐penetrating, surface‐engineered exosomes could be functionalized as active agent nanocarriers. The functionalized exosomes can be employed in five main cancer treatment strategies, including chemotherapy, gene therapy, immunotherapy, phototherapy, and vaccine development.^[^ ^79^ ^]^ Peptides, nucleic acids, antibodies, lipids and chemical reagents, e.g., PEG conjugated with exosomes are considered as modifying agents.^[^ ^79^ ^]^ They could attach to tumor vessels and cell receptors to deliver more dosage of active agents to tumors than healthy cells.^[^ ^52^ ^]^ For example, polypeptide‐modified exosomes were applied to deliver anti‐apoptotic Bcl‐2 antisense oligonucleotide G3139, which enhanced cell penetrating.^[^ ^85^ ^]^ Exosomes could be modified by PEG to improve the circulation time in the blood and increase the accuracy of targeted cancer cells.^[^ ^86^ ^]^ They are also used as a coating agent for metal‐organic framework nanoparticles to impede leakage of the anti‐cancer drug, protect the nanocarrier from degraded enzymes and hide them from the immune system.^[^ ^58^ ^]^ Tumor cell‐derived exosomes can significantly inhibit tumor growth in ectopic and orthotopic hepatocellular carcinoma models.^[^ ^24^ ^]^ Exosomes secreted by MSCs, have remarkable antitumor features and are able to target tumor cells through proteasome transfer.^[^ ^87^ ^]^

These outcomes provided that the unique features of exosomes make them promising for tumor‐targeted delivery and cancer therapeutics. More research is required to regulate the immunological and therapeutic aspects.

#### 2.1.2 Cardiovascular Disease

**Figure 4:**
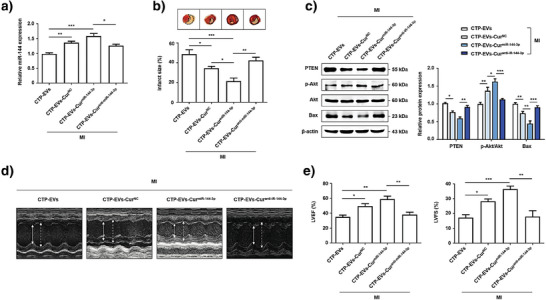
A) Assessing the miR‐144‐3p expression in mice heart tissues using qRT‐PCR; *n* = 3 per group. B) Representative photos of heart sections using TTC‐stain and quantified data quantifying the size of myocardial infarction one week after MI in mice injected with CTP‐EVs, CTP‐EVs‐CurNC, CTP‐EVs‐CurmiR‐144‐3p, or CTP‐EVs‐Curanti‐miR‐144‐3p; *n* = 5 per group. C) Representative blots and quantified data showing protein expression in the indicated groups. D,E) Representative M‐mode images (E) and quantified data showing mice injected with CTP‐EVs‐Cur miR‐144‐3p have higher EF% and FS% compared to CTP‐EVs‐Cur anti‐miR‐144‐3p‐injected mice Reproduced with permission,^[^ ^91^ ^]^ 2021 Elsevier.

Another leading cause of mortality and morbidity is CVDs, mainly myocardial infarction (MI), heart failure, cardiac fibrosis, atherosclerosis, ischemia‐reperfusion injury, etc., associated with high blood pressure, progressive inflammation, and chronic heart disorder. In 2019, ≈18.6 million individuals died from CVD worldwide.^[^ ^88^ ^]^ The common approaches for CVD treatment are medications, invasive surgery, or their combination. In traditional CVD surgery, the damaged cardiac area has been operated on to prevent thrombosis, and an artificial pacemaker has been implanted to treat arrhythmia, which has a negative effect on patient compliance for a lifetime. The primary goals of CVD therapy via medications are reducing the growth and thickness of atherosclerotic plaques, restoring the natural blood flow through or around damaged vasculature, preventing clot formation, and inhibiting frequent cardiovascular shocks.^[^ ^89^ ^]^ In recent years, with advancements in nanomedicine, a novel therapeutic approach for CVDs and cardiac regeneration has been designed. Nanocarriers provide a platform to enhance targeted delivery and accelerate healing mechanisms by passing the endothelium of blood vessels regarding CVDs. However, toxicity, protein corona formation, biological safety, off‐target aggregation, weak tissue permeability and circulation time affect the efficacy of synthetic nanoparticles as active agent nanocarriers.^[^ ^89^, ^90^ ^]^ To overcome these challenges by considering the novel therapeutic approaches for CVDs, exosomes could be an alternative and function as cargoes of CVD‐related active agents (**Figure** **4**), to target cells for their cardiac regenerative and protective potential.^[^ ^59^ ^]^

Exosomes derived from MSC, cardiac‐derived progenitor cells, embryonic stem and cells adipose‐derived stem cells, carry various RNAs, such as miR‐144, miR‐21, miR‐22, miR‐23 and miR‐24, which serve as modifiers and cardioprotective active agent for target cells in CVDs.^[^ ^92^, ^93^ ^]^ Particularly, MSC‐derived exosomes are cholesterol‐rich phospholipids that afford the protection of their contents.^[^ ^94^ ^]^ Exosomal miR‐31 from adipose‐derived stem cells promotes blood perfusion and angiogenesis in both mouse ischemic hindlimb and heart.^[^ ^95^ ^]^ It has been observed that the cardioprotection against ischemia‐reperfusion injury in vivo could be mediated by increasing exosome cargoes containing miR‐144.^[^ ^96^ ^]^ According to a recent study, co‐delivery of miR‐144 and curcumin through cardiac targeting peptide exosomes shows a great cardioprotective effect and provides a promising nanomedicine for MI therapy.^[^ ^91^ ^]^ Circulating exosome‐originated miR‐126 has been found to play a role in cardiac repair during acute MI.^[^ ^97^ ^]^ Additionally, exosomes derived by cardiac progenitor cells, could promote angiogenesis and cardiac cell survival by releasing miR‐210, miR‐132, and miR‐146a‐3p in acute MI.^[^ ^98^ ^]^ Moreover, another in vivo experiment confirmed the significant effect of miR‐21 originating from exosomes on preventing scar formation in the area of MI and further improving cardiac function. However, it is also reported that high expression of miR‐21 in exosomes originating from human embryonic kidney cells could improve the apoptosis of cardiac myocytes and endothelial cells; therefore, the role of exosome‐released miR‐21 depends highly on the context.^[^ ^99^ ^]^ Furthermore, MSC‐derived exosomes are most widely used for cardiac repair and regeneration. Intravenous administration of these exosomes in vivo significantly increased cardiac function and angiogenesis in MI.^[^ ^100^ ^]^ It is also reported that MSC‐derived exosomes enriched in miR‐19a positively impact the recovery of cardiac function and reduction in infarct size in acute MI.^[^ ^101^ ^]^ Lai et al. designed a triple hybrid cellular nanovesicle to treat myocardial ischemia/reperfusion (I/R) injury, which results from reperfusion therapy for acute MI.^[^ ^102^ ^]^ Myocardial ischemia/reperfusion (I/R) injury results in an increase in necrotic cells aggregation and influences the inflammatory response.^[^ ^102^ ^]^ Their nanovesicles system is composed of cell‐derived nanovesicles with high expression of high‐affinity SIRPα variants (SαV‐NVs), exosomes derived from mesenchymal stem cells (MSCs), and platelet‐derived nanovesicles (PLT‐NVs).^[^ ^102^ ^]^ The function of SαV‐NVs, exosomes, and PLT‐NVs is to provide the vesicles to bind to the CD47 protein that plays a major role in allowing the necrotic cells to escape the phagocytosis process of macrophages, to regulate the cardiac inflammatory cytokines, and to inherit the ability of platelets to adhere to wounded vasculatures, respectively.^[^ ^102^ ^]^ The authors conducted a comprehensive evaluation of cardiac histopathology and function after 3 weeks of I/R surgery, followed by the triple hybrid cellular nanovesicles. They found a significant reduction in cardiac fibrosis and an increase in left ventricular ejection fractions compared to the negative control group.^[^ ^102^ ^]^ Lai et al. triple hybrid cellular nanovesicles show a promising mechanism for decreasing cardiac injury due to their ability to integrate CD47 blocking and inflammation regulation treating mechanisms.

Nevertheless, the delivery of exosomes to the heart is one of the main challenges. Because of liver accumulation of exosomes, the intravenous administration for cardiac delivery is not efficient. The other routes of administration, such as intramyocardial exosome injection, are more effective, but they need invasive procedures.^[^ ^103^ ^]^ Moreover, the therapeutic role of exosomes in CVDs is at an early stage and clinical studies, have been carried out in animal experiments and in multiple cardiac‐related cells. It is absolutely essential that exosome‐based experiments on other mammals and human should be performed as well as novel surface‐engineered exosomes should be explored to address these barriers in the future.^[^ ^96^, ^103^ ^]^

#### 2.1.3 Orthopedic Disease

**Figure 5:**
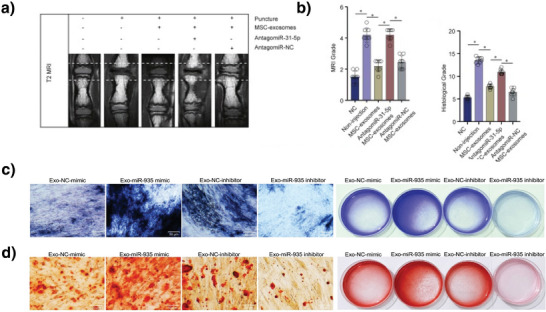
A) MRI of the rat tails in different groups. B) The scores of MRI in different groups. A,B) Show that the MRI score of the MSC‐exosome group was significantly lower compared to the non‐injection group.^[^ ^105^ ^]^ C) ALP staining for cell activity (scale bar = 50 µm) shows that ALP staining is stronger in osteoblasts co‐cultured with Exo‐miR‐935 compared to the one co‐cultured with Exo‐miR‐935 inhibitor. D) Detection of calcified nodules by alizarin red staining shows that osteoblasts co‐cultured with Exo‐miR‐935 mimic treatment enhance the deposition of minerals. Reproduced with permission,^[^ ^106^ ^]^ 2021 Elsevier.

The novel therapeutic approach for bone‐related disease is another urgent and vital area of research, which has been considered a target for exosome‐based treatments (**Figure** **5**). Orthopedic diseases, particularly general bone‐related diseases comprising degenerative intervertebral disc degeneration (IVDD), musculoskeletal disorders, osteoarthropathy, osteoarthritis (OA), and osteoporosis, have a significant effect on healthcare systems. Treatment of bone and cartilage defects is also a great challenge in orthopedic surgery.^[^ ^11^, ^104^ ^]^ In addition, the synthetic drug delivery systems and biomaterials usually fail to transfer sufficient concentration of active agents in targeting sites and suffer from the lack of long plasma half‐life time and side effects due to vascular and cellular obstacles, mainly comprising complex bone extracellular matrix that hinders the diffusion, the phagocytosis by macrophages, and unspecific uptake by hematopoietic or MSC.^[^ ^54^ ^]^ Consequently, safe and efficient medications for bone‐related disorders need to be developed.

Exosomes could be a breakthrough for bone‐issue therapy. The specific miRNAs associated with osteoporosis, OA, osteoblasts differentiation, and IVDD could be shut down by exosomes, especially derived from MSCs to the targeting sites. MSC‐derived exosomes are efficacious for bone regeneration in animal models of bone defects and diseases such as osteonecrosis and osteoporosis. They also could promote new bone formation with supporting vasculature and display improved morphological, biomechanical, and histological outcomes.^[^ ^107^ ^]^ They have been suggested as a promising alternative therapy for OA.^[^ ^108^ ^]^ Moreover, exosomes secreted by bone cells, such as osteoclasts, osteoblasts, and their precursors, can adjust bone remodeling by delivering biologically active molecules to target cells.^[^ ^109^ ^]^ A recent in vivo study shows that the bone marrow MSC‐derived exosomes enriched by miR‐935 promote osteoblast proliferation and differentiation.^[^ ^106^ ^]^ Another in vivo study revealed, that the mussel‐inspired highly adhesive hydrogel with encapsulated bone marrow MSC‐derived exosomes promotes bone marrow MSCs proliferation and differentiation, thereby accelerate the cartilage defect regeneration.^[^ ^110^ ^]^ It has also been reported that after in vivo experiments, MSC‐exosomes enriched by miR‐31‐5p ameliorate IVDD, although by a low level of miR‐31‐5p, the protective effects are inhibited.^[^ ^105^ ^]^ These extraordinary findings show the great potential of exosomes in the treatment and regeneration of bone disorders.

Considering that a wide range of molecules perform the bone regeneration processes, the bone targeting mechanism should be improved by employing exosomes. As a result of advancements in the engineering and technology of exosomes, they could illustrate more advantages in bone‐related diseases in the future.

#### 2.1.4 Neurological Diseases

One in four people worldwide may have a mental or neurological disorder at some point in their lives, according to statistics. These problems affect over 450 million people globally, making neurological disorders one of the main causes of illness and disability.^[^ ^111^ ^]^ Currently, AD affects 5 million Americans, followed by PD affects 1 million. The prevalence of ND is anticipated to increase to 12 million in 2050 in the United States, which represents a serious public health issue.^[^ ^112^ ^]^

The phrase “neurodegeneration” could be described as the detriment of structure, function, or irreversible loss of neuronal cells, leading to a pathological condition. Therefore, NDs demonstrate a sort of neurological disorders that are characterized by cognitive and behavioral dysfunctions of the nervous system and the chronic progressive loss of neurons,^[^ ^113^ ^]^ associated with aging, genetics, environmental factors, etc.,^[^ ^114^ ^]^ mainly including AD, PD, multiple sclerosis (MS), amyotrophic lateral sclerosis (ALS), Huntington's disease (HD) and the prion disease. There are specific proteins associated with different types of NDs comprising the infectious scrapie‐associated prion protein (PrP^Sc^) in prion disease, the amyloid‐β (Aβ) and protein tau (p‐Tau) in AD, α‐synuclein (α‐syn) in PD, mutant huntingtin proteins (mHtt) in HD, and superoxide dismutase 1 (SOD1), TDP‐43, FUS/ TLS, and C9ORF72 in ALS.^[^ ^2^, ^9^, ^32^, ^113^, ^115^ ^]^

AD has been known as chronic age‐related ND associated with cognitive decline and dementia.^[^ ^116^ ^]^ Neurotic plaques, senile amyloid plaques and neurofibrillary tangles (NFTs) are the main neuropathological features of AD,^[^ ^117^ ^]^ which leads to the histopathological alteration in the AD brain that can be distinct into two processes; primarily, the formation of plaques by deposition of Aβ protein and secondly, the originating of intercellular NFTs from paired helical filament (PHF) of microtubule‐associated hyperphosphorylated p‐Tau.^[^ ^9^, ^117^ ^]^ It has been reported that exosomes have a significant role in spreading and accumulating toxic proteins such as Aβ and p‐Tau by endosomal pathway and axonal transport.^[^ ^14^, ^118^, ^119^ ^]^ There are also other AD‐associated proteins and peptides, such as amyloid precursor protein (APP), APP C‐terminal fragment (source of Aβ peptides), or APP intracellular domain, which are released by exosomes to the outside of cells.^[^ ^120^ ^]^ PD is the second most prevalent chronic ND, occurring in sporadically or familial form and characterized by motor symptoms such as bradykinesia (slowness of movement), rigidity, resting tremor, and postural instability as well as non‐motor symptoms, e.g. olfactory dysfunction, sleep disturbances, constipation and depression.^[^ ^121^, ^122^ ^]^

Treatment and early diagnosis of NDs are major issues for biomedical researchers because the trend of the prevalence of ND increases consistently, closely associated with intense psychological pressure and economic burden to patients, families, and the healthcare system. The available treatments are few or not effective, and the lack of treatment options for NDs requires the development of novel therapeutic approaches with the utmost efficacy and chance of success.^[^ ^123^ ^]^ Therefore, reliable and easily accessible biomarkers and effective therapeutic options are crucially needed for NDs.

To improve and extend the quality of life for ND patients, especially among older adults, developing new potential therapies against NDs is a crucial prerequisite. Furthermore, it is difficult to elucidate the exact reason for the progressive neuronal loss because of the complexity of NDs. Not only are CNS disorders protected by the BBB, which physiologically maintains homeostasis by shielding CNS against most of the peripheral blood supplies, but also BBB acts as a barrier for the delivery of therapeutic agents^[^ ^124^ ^]^ Considering the fact that it remains a lack of effective therapeutic agents due to the obscure cause of neuronal death and the impeded early diagnosis of neurodegenerative diseases,^[^ ^125^ ^]^ there is a need for novel approaches to overcome these challenges. The complexity of intervention in ND is challenging, but an exosome‐based delivery approach could have a positive neuroprotective effect and transport adequate drug concentration to the brain cells to achieve the therapeutic goals.^[^ ^36^, ^126^ ^]^ In addition, because of the specific features of exosomes, e.g., RNA transport capacity, long‐time circulation, and ability to cross BBB, the site‐specific‐derived exosomes have a great potential for use as a nanocarrier to transport therapeutic agents to brain cells and degrade the toxic proteins.^[^ ^118^ ^]^ Thus, they could function as an alternative possibility in treating NDs and provide a novel approach for long‐term ND treatment.

Exosome‐based treatment has emerged as a promising therapeutic approach for various NDs, including AD, PD, MS, ALS, and stroke.^[^ ^127^, ^128^, ^129^, ^130^, ^131^ ^]^ Exosomes derived from different cell types, such as MSCs, neural stem cells (NSCs), and adipose‐derived stem cells (ADSCs), have shown neuroprotective effects in neurodegenerative diseases through modulation of various signaling pathways, including the phosphoinositide 3‐kinase (PI3K)/protein kinase B (Akt)/mammalian target of rapamycin (mTOR) pathway.^[^ ^127^ ^]^ For instance, MSC‐derived exosomes have been shown to improve cognitive function in AD models by activating the PI3K/Akt/GSK‐3β pathway and reducing neuroinflammation.^[^ ^127^ ^]^ NSC‐derived exosomes have been found to promote functional recovery in stroke models by activating the CREB/BDNF/TrkB pathway.^[^ ^127^ ^]^ ADSC‐derived exosomes have been shown to attenuate neuronal apoptosis and inflammation in spinal cord injury models by activating the PI3K/Akt pathway.^[^ ^127^ ^]^ In addition to their therapeutic effects, exosomes have been investigated as drug delivery vehicles for the treatment of neurological diseases.^[^ ^129^ ^]^ They have been loaded with various drugs and therapeutic molecules, such as miRNAs and growth factors, to enhance their therapeutic potential.^[^ ^129^ ^]^ For example, exosomes derived from MSCs overexpressing miRNA‐133b or enriched with the miRNA‐17‐92 cluster were found to significantly improve brain plasticity and functional outcomes in stroke animals.^[^ ^128^ ^]^

Despite the promising results, there are still challenges in the use of exosomes as therapeutic agents, including difficulties in characterization, lack of controlled drug release mechanisms, and inefficient isolation methods.^[^ ^127^ ^]^ Different isolation techniques can be used to obtain exosomes, including ultracentrifugation, polymer‐based precipitation, immunoaffinity capture, and size‐based isolation.^[^ ^130^ ^]^ Each technique has its advantages and limitations, and the choice of method depends on the specific requirements of the study or therapeutic application.^[^ ^130^ ^]^

### 2.2 Perspectives and Challenges of Therapeutic Applications of Exosomes

Besides the aforementioned advantages of exosome‐based drug delivery systems, there are also many challenges and obstacles. A serious issue that is currently restricting the clinical application of exosomes is a lack of quality control and standardized procedures.^[^ ^22^ ^]^ Due to the significant complexity of exosome systems, their role in overall disease and health conditions, a deep understanding of their nature and long‐term safety and therapeutic effects should be considered in future studies. In addition, a comprehensive understanding of determinant attributes leading to selective‐cell uptake of exosomes and factors influencing their intracellular disposition should be investigated.^[^ ^132^ ^]^ Moreover, isolation and maintenance of exosomes with high purity on a large scale are essential if exosomes must be utilized as drug delivery carriers for mass production. Furthermore, the attachment of ligands on the surface of exosomes through chemical conjugation must be examined for targeting drug delivery in terms of safety and efficacy. Exosomes consist of heterogeneous components that exhibit different cellular functions. If inappropriate donor cells are chosen to derive exosomes they may show immunogenicity effects.^[^ ^133^ ^]^ Therefore, the choice of exosome origin plays a critical role for particular disease and specific targeting exosome‐based nanocarrier. Undoubtedly to overcome these challenges and guarantee the safety and efficacy of exosome‐based therapeutics, as well as transition through pre‐clinical to clinical application, further investigation and research are necessary.

## 3 Chronic Disease Diagnosis via Exosomal Biomarkers

**Figure 6:**
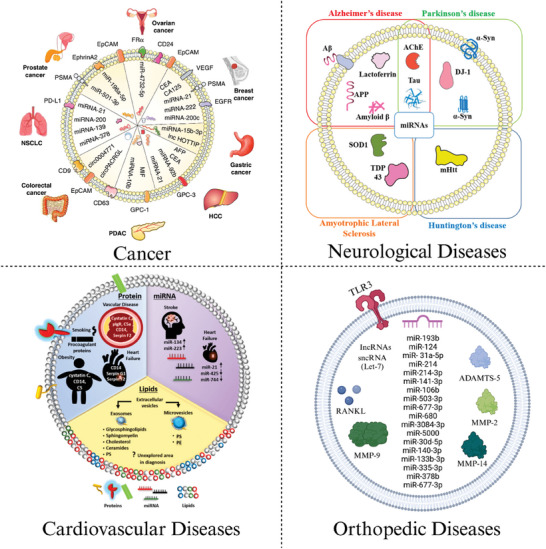
The potential of exosomal biomarkers for different chronic diseases (created using Biorender.com). Reproduced with permission,^[^ ^134^ ^]^ 2022 Authors. Reproduced under the terms of the CC‐BY license.^[^ ^9^, ^135^ ^]^ copyrights 2019 and 2021.

The early and accurate diagnosis of chronic diseases stands as a pivotal challenge in modern healthcare. Amidst the evolving landscape of diagnostic methodologies, the use of exosomal biomarkers has emerged as a promising frontier (**Figure** **6** and **Table** **3**). From cancer to NDs, this section highlights the significant strides being made in harnessing exosomes as non‐invasive and sensitive tools for early disease detection and monitoring, ultimately advancing the prospects of personalized medicine and improved patient outcomes.

**Table 3:** Key exosomal biomarkers in chronic diseases.

Disease	Origin of exosomes	Suggested exosomal biomarker(s)	Outcomes/diagnosis
Cancer	Plasma	Exosomal protein glypican1 protein	In patients with breast and pancreatic tumors^[^ ^140^ ^]^
		Exosomal protein glypican1 protein miR‐96‐5p and miR‐149	Colorectal cancer^[^ ^141^ ^]^
		Long non‐coding RNA (lnRNA) (H19)	Breast cancer^[^ ^142^ ^]^
Cardiovascular	Macrophages	miRNA‐155	Promote fibroblast inflammation and decrease fibroblast proliferation^[^ ^148^ ^]^
	Mature dendritic cells	Activating the membrane TNF‐α‐mediated NF‐kB pathway	Enhance endothelial atherosclerosis and inflammation formation of atherosclerosis^[^ ^150^ ^]^
	Plasma	miR‐30e and miR‐92a	In patients with coronary atherosclerosis repressing a major regulator factor of phospholipid homeostasis and cellular cholesterol^[^ ^151^ ^]^
		C1q subcomponent subunit A (C1QA), complement C5 (C5), apolipoprotein C‐III (APOCC3), apolipoprotein D (APOD), platelet basic protein (PBP), and platelet glycoprotein Ib alpha chain (GP1BA)	In patients with myocardial infarction^[^ ^152^ ^]^
		p53‐responsive miRNAs (miRNA‐34a, miR‐192, and miR‐194)	In heart failure^[^ ^153^ ^]^
Orthopedic	Plasma	miR‐193b	Reduced in patients with osteoarthritis^[^ ^154^ ^]^
		Platelet‐derived exosomes	Promoting the progression of joint diseases by inducing the production of pro‐inflammatory factors^[^ ^155^ ^]^
		lncRNAs	Increased in both early‐stage and late‐stage osteoarthritis^[^ ^156^ ^]^
		lncRNAs and miRNAs derived from exosomes of immune‐related cells (such as T cells, macrophages, dendritic cells, etc.)	In mediating inflammatory responses associated with bone diseases^[^ ^157^ ^]^
	Synovial fluid	miR‐124	Modulating macrophage activity in joint diseases and inflammation^[^ ^158^ ^]^
		miRNAs	Modulate the autophagic process in macrophages influence the progression of rheumatoid arthritis Early and accurate diagnosis of OA^[^ ^159^ ^]^
		Exosomes from fibroblast‐like synoviocytes	In patients with rheumatoid arthritis can lead to joint damage^[^ ^156^ ^]^
	Serum	Level of exosomes	Decreased level of exosomes useful in detection of early‐stage osteonecrosis of the femoral head^[^ ^160^ ^]^
		TLR3 protein	Propagating inflammation by inducing the activation of NF‐κB pathway and the production of type I IFN in patients with rheumatoid arthritis^[^ ^155^ ^]^
AD	Plasma	Aβ	Early diagnosis of AD^[^ ^161^ ^]^
		Tau	AD diagnosis,^[^ ^119^ ^]^ interaction of Aβ and Tau^[^ ^162^ ^]^
		miR‐342‐3p, miR‐141‐3p, miR‐342‐5p, miR‐23b‐3p, miR‐24‐3p, miR‐125b‐5p, and miR‐152‐3p	Associated with AD^[^ ^163^ ^]^
		Growth associated protein 43 (GAP43), neurogranin, synaptosome associated protein 25 (SNAP25), and synaptotagmin 1	Associated with AD^[^ ^164^ ^]^
		Proteins: A0A0G2JRQ6, C1QC, CO9, GP1BB, RSU1, and ADA10)	Associated with AD^[^ ^165^ ^]^
	CSF	miR‐29c, miR‐136‐3p, miR‐16‐2, miR‐331‐5p, miR‐132‐5p, & miR‐485‐5p	Involved in amyloid‐beta accumulation, tau‐dependent toxicity, inflammation, and neuronal death^[^ ^163^, ^164^ ^]^
PD	Plasma	α‐syn	Significantly higher^[^ ^166^, ^167^ ^]^ Related to severity of PD^[^ ^167^ ^]^ Early detection of PD^[^ ^32^ ^]^
		Clusterin, C1r, and ApoE A1	Significantly lower at HY stage II and III^[^ ^168^ ^]^ Level of ApoE A1 related to the severity of PD^[^ ^168^ ^]^
		Exosomes derived from neuron, astrocyte and oligodendrocyte	Significant increase by neuron‐derived exosomes in mild PD^[^ ^169^ ^]^ Severity monitoring of PD, MSA and PSP by exosomal oligodendrocyte^[^ ^169^ ^]^
		AChE	Significantly lower^[^ ^170^ ^]^ Negative correlation with disease severity^[^ ^170^ ^]^ Likely proper for early diagnosis^[^ ^170^ ^]^
		DJ‐1	Significantly higher^[^ ^166^ ^]^
		miR‐331‐5p,^[^ ^171^ ^]^ Let‐7e‐5p^[^ ^172^ ^]^	Higher^[^ ^171^, ^172^ ^]^
		miR‐505^[^ ^171^ ^]^	Lower^[^ ^171^ ^]^
		lnc‐MKRN2‐42:1 RNA	positively correlated with the severity of dyskinesia and dysarthria^[^ ^173^ ^]^
	CSF	α‐syn	Higher^[^ ^174^ ^]^/lower^[^ ^175^ ^]^
		Let‐7f‐5p, miR‐125a‐5p, miR‐10b‐5p and miR‐151a‐3p,^[^ ^176^ ^]^ miR‐153, miR‐409‐3p and miR‐10a‐5p^[^ ^177^ ^]^	Higher^[^ ^176^, ^177^ ^]^ Efficiently distinguish early‐stage PD from non‐PD^[^ ^176^ ^]^
		miR‐27a‐3p, miR‐423‐5p, miR‐151a‐3p and miR‐22‐3p,^[^ ^176^ ^]^ miR‐1 and miR‐19b‐3p^[^ ^177^ ^]^	Lower^[^ ^176^, ^177^ ^]^ Efficiently distinguish early‐stage PD from non‐PD^[^ ^176^ ^]^
		Tau	Significantly higher^[^ ^178^ ^]^
	Serum	α‐syn	Lower^[^ ^179^ ^]^/higher^[^ ^180^ ^]^
		miR‐24 and miR‐195,^[^ ^181^ ^]^ miR‐29c,^[^ ^182^ ^]^ miR‐29a^[^ ^183^ ^]^	Higher^[^ ^181^, ^183^ ^]^/Significantly higher^[^ ^182^ ^]^
		miR‐19b,^[^ ^181^ ^]^ miR‐21‐3p, miR‐22‐3p and miR‐223‐5p, miR‐425‐5p, miR‐21‐3p, and miR‐199a‐5p^[^ ^184^ ^]^	Lower^[^ ^181^ ^]^ Discrimination of PD from non‐PD^[^ ^184^ ^]^ Distinguish PD from PSP^[^ ^184^ ^]^
	Urine	DJ‐1	Significantly higher in male^[^ ^185^ ^]^
		Ser(P)‐1292 LRRK2	No difference^[^ ^185^ ^]^/Higher^[^ ^186^, ^187^ ^]^
	Saliva	α‐syn	Significantly higher^[^ ^188^ ^]^

### 3.1 Cancer

Nowadays, several diagnostic methods are widely used in cancer diagnosis, however, these techniques, such as imaging tools and biopsies, are often expensive, invasive and detecting tumors at early stages is still challenging. In fact, there is a strong correlation between how early the disease is diagnosed and the survival rates. For example, the survival rates when cancer is diagnosed at early stages are much higher than at late stages.^[^ ^137^ ^]^ This is due to the fact that early diagnosis of cancer enhances the successfulness of the clinical intervention, such as treating the tumor with a milder therapeutic regimen or removing the tumor surgically.^[^ ^137^ ^]^ Thus, diagnosing cancers at early stages in a non‐invasive and cost‐effective manner is needed to enhance treatment efficacy, decrease treatment resistance, and increase cancer survival rates.

Several efforts have been made to reveal biomarkers that provide information about tumor development and progression. Researchers express great enthusiasm for substituting traditional solid biopsy with liquid biopsy to detect early‐stage tumors.^[^ ^138^ ^]^ This is for many reasons: (I) liquid biopsy method is non‐invasive compared to traditional solid biopsy since it can be easily obtained. (II) it is more accurate and sensitive and provides more information about the tumor and its progression.^[^ ^138^ ^]^

Liquid biopsy is the collection and examination of biofluids, including circulating tumor cells (CTCs), nucleic acids (e.g., cell‐free DNA), and exosomes.^[^ ^134^ ^]^ Exosomes are superior in all the compartments of liquid biopsy since they can be found in all biofluids (e.g., blood, urine, and saliva). Also, exosomes are stable and protect their contents from degradation by encapsulating the interior space with a phospholipid bilayer.^[^ ^134^ ^]^ Thus, using exosomes in the last decade as a biomarker holds many promises in tackling the diagnosis of multifactorial diseases, in general, and cancer, in particular.^[^ ^139^ ^]^

Melo et al. hypothesized that exosomes specifically the ones that are secreted from cancer cells contain glypican1 protein (GPC1); thus, exosomal GPC1 can be utilized as a biomarker for breast and pancreatic cancer.^[^ ^140^ ^]^ Their hypothesis is based on the fact that GPC1 is a membrane‐bound protein that is overexpressed in breast and pancreatic tumors. They isolated exosomes from plasma samples of 32 breast cancer patients, 190 pancreatic ductal adenocarcinoma patients (PDAC), and 100 healthy controls (HC).^[^ ^140^ ^]^ The authors contend that GPC1 was highly expressed in exosomes derived from plasma of 75% of breast cancer patients compared to healthy controls. Moreover, exosomal GPC1 derived from all PDAC patients (100%) was significantly higher than that derived from healthy controls.^[^ ^140^ ^]^ This is a proof of concept that exosomal GPC1 has the potential to provide a promising biomarker specifically for pancreatic cancers. The authors compared the specificity and sensitivity of exosomal GPC1 to carbohydrate antigen 19‐9 (CA19‐9), a PDAC standard biomarker, where they found that exosomal GPC1 was able to differentiate PDAC patients not only from healthy controls but also from benign pancreatic disease with a specificity and sensitivity of 100%, whereas CA19‐9 biomarker showed ≈20%–30% less sensitivity and specificity.^[^ ^140^ ^]^ Also, exosomal GPC1 was able to detect the disease in all stages (i.e., Stage I, II, III, IV) in specificity and sensitivity of 100%, indicating its ability to be utilized as a biomarker for the disease in all its stages and its potential to detect the tumor in its early phases.^[^ ^140^ ^]^ Another study explored the ability of exosomal GPC1 to act as a biomarker for colorectal cancer (CRC).^[^ ^141^ ^]^ The authors found that the expression of GPC1 in exosomes obtained from the plasma of CRC was significantly higher than that isolated from the plasma of HC. Also, they found that there was a significant difference in the expression of miR‐96‐5p and miR‐149 in the exosomes obtained from the plasma of CRC patients and HCs.^[^ ^141^ ^]^

Zhong et al. analyzed the expression of long non‐coding RNA (lnRNA) (H19) in the exosomes derived from plasma samples obtained from 50 breast cancer patients, 50 benign breast disease, and 50 healthy controls.^[^ ^142^ ^]^ The authors mentioned that exosomal H19 was able to differentiate not only breast cancer patients from benign breast disease and HCs with a specificity and sensitivity of 70.6% and 87.0%, respectively.^[^ ^142^ ^]^ Moreover, they stated that exosomal H19 showed a significantly higher area under the curve (AUC), sensitivity, and specificity compared to the traditional breast cancer biomarkers, i.e., cancer antigen 15‐3 (CA15‐3) and carcinoembryonic antigen (CEA).^[^ ^142^ ^]^

Several studies have investigated the ability of exosomes to serve as biomarkers for different types of cancers by analyzing their content (e.g., DNAs, miRNAs, proteins). These studies show that exosomes hold promise as reliable, non‐invasive biomarkers for diagnosing cancers at early stages and throughout disease progression.^[^ ^143^, ^144^ ^]^ Despite attempting to design diagnostic devices based on exosomes that achieve a successful cancer diagnostic platform, only a limited number of exosomes based devices, e.g. ExoDx Prostate (IntelliScore) and ExoDxLung (ALK) have been successfully translated from laboratory benches to the market. The former device is the first device to detect prostate cancer by analyzing the expression of three exosomal biomarkers, namely prostate cancer gene 3 (PCA3), erythroblast transformation‐specific related gene (ERG), and SAM pointed domain containing ETS transcription factor (SPDEF).^[^ ^145^ ^]^ A score of more than 15.6 indicates an increased risk of developing high‐grade prostate cancer.^[^ ^145^ ^]^ ExoDx Lung(ALK) is another diagnostic device that has been approved to be utilized in clinics. It is a prognostic test that detects the risk of developing lung cancer by analyzing the expression of RNA in exosomes derived from plasma, namely EML4‐ALK, which is the outcome of the fusion of two genes known as echinoderm microtubule‐associated protein‐like 4 (EML4) and anaplastic lymphoma kinase (ALK).^[^ ^145^ ^]^

To translate more diagnostic devices into the market and to achieve the potential promises of exosomes as a source of reliable biomarkers for cancer, it is crucial to address several challenges. First, there is still a significant demand for highly effective and sensitive quantitative approaches to identify tumor‐derived exosomes in a cost‐effective and timely manner. Second, there is currently a lack of a standardized exosome separation method that can reliably extract exosomes with high purity and yield. Overcoming such challenges will ultimately result in accelerating the translation of exosome cancer diagnostic platforms from lab benches to the market.

### 3.2 Cardiovascular Disease

According to the WHO, CVDs have been classified as the leading cause of death globally.^[^ ^146^ ^]^ Several diseases can be classified under the umbrella of CVDs, including myocardial infarction, stroke, and heart failure.

The cardiovascular system (CVS) consists of several cells, such as cardiomyocytes, endothelial cells, lymphatic cells, atherosclerosis cells, and vascular smooth muscle cells.^[^ ^147^ ^]^ Several researchers have found that exosomes also play a crucial role in the communication between different types of CVS cells and maintain the physiological processes of the system. These biological vesicles carry valuable information that reflects the physiological condition (e.g., cardiac development and myocardial angiogenesis) and pathological condition of their cellular origin. For instance, in cardiac tissue injury cases, activated macrophages produce and release exosomes enriched with miRNA‐155, which have been demonstrated to promote fibroblast inflammation and decrease fibroblast proliferation directly.^[^ ^148^ ^]^ Owing to the significant contribution of exosomes in the intercellular communication between the different types of cells in the CVS, research has been done to analyze the content of exosomes derived from the CVS and investigate their ability to act as a biomarker for several CVDs. The following discussion will explore the potential roles of exosomal content in diagnosing CVDs.

One of the most common types of CVD is coronary artery disease (CAD).^[^ ^149^ ^]^ CAD jeopardizes the health of millions of people every year worldwide.^[^ ^149^ ^]^ CAD is mainly caused by atherosclerosis, which is made up of the accumulation of lipids in the inner lining of the large arteries that supply blood to the heart.^[^ ^149^ ^]^ The reason behind the formation of atherosclerosis is related to multiple factors, including endothelial dysfunction, inflammation, and oxidative stress.^[^ ^149^ ^]^ In fact, it has been shown that exosomes have a major indirect contribution to the formation of atherosclerosis. For instance, exosomes released from mature dendritic cells enhance endothelial atherosclerosis and inflammation through the membrane TNF‐α‐mediated NF‐kB pathway.^[^ ^150^ ^]^

Exosomes have shown mounting promise in the prognosis and diagnosis of CAD via analyzing the exosomal contents, including proteins, miRNA, RNA, and DNA. In 2019, Wang et al. investigated the ability to discover novel coronary atherosclerosis biomarkers by analyzing the content of exosomes obtained from the plasma of 42 individuals diagnosed with coronary atherosclerosis.^[^ ^151^ ^]^ The authors identified 9 miRNAs candidates which are highly related to the disease.^[^ ^151^ ^]^ For instance, they found that the expression of miR‐30e and miR‐92a was highly upregulated in the exosomes obtained from the plasma of individuals diagnosed with coronary atherosclerosis compared to that obtained from the plasma of healthy controls.^[^ ^151^ ^]^ Furthermore, the authors studied the relationship between the two miRNAs, miR‐30e and miR‐92a, and the pathogenesis of the disease, where they found that the two miRNAs have a major impact on repressing a major regulator factor of phospholipid homeostasis and cellular cholesterol, known as ATP binding cassette (ABC)A1.^[^ ^151^ ^]^

One of the common consequences of CAD is myocardial infarction (MI), commonly referred to as heart failure (HF). MI is responsible for the majority of CAD‐related deaths. Currently, the expression of proteins, like creatinine kinase MB and troponin in blood circulation, is used as a biomarker for MI. In fact, the current biomarkers are a major reason for the decline in the mortality statistics from MI. This is because they allow earlier recognition of the disease and enhance the effectiveness of the clinical intervention, such as coronary artery bypass surgery (CABG). For example, troponin levels in AMI patients rise as early as 3.5 h following the beginning of chest pain. However, researchers are still striving to discover new early biomarkers with high sensitivity and specificity to reduce the mortality of MI further. Cheow et al. performed a proteomic analysis for exosomes obtained from the plasma of MI patients.^[^ ^152^ ^]^ They found an increase in the expression of six exosomal proteins in MI patients, including complement C1q subcomponent subunit A (C1QA), complement C5 (C5), apolipoprotein C‐III (APOCC3), apolipoprotein D (APOD), platelet basic protein (PBP), and platelet glycoprotein Ib alpha chain (GP1BA).^[^ ^152^ ^]^

Another study found that in MI, cardiomyocytes are exposed to a hypoxic condition that triggers the cells to secrete exosomes with a high expression of tumor necrosis factor α (TNFα), which can also act as a promising biomarker for the disease. Matsumoto et al. explored the expression of 377 miRNAs in the plasma of individuals diagnosed with heart failure and compared it to their expression in the plasma of healthy controls.^[^ ^153^ ^]^ The authors found that the circulating levels of p53‐responsive miRNAs, such as miRNA‐34a, miR‐192, and miR‐194, rise significantly in the plasma of individuals diagnosed with heart failure.^[^ ^153^ ^]^ Further, they found that the three miRNAs were released into the plasma via exosomes since they were enriched in the exosomes derived from the plasma of the individuals diagnosed with heart failure, and their expression was significantly higher than their expression in the exosomes derived from the plasma of healthy controls. The study by Matsumoto et al. demonstrates that exosomes can be used as a reliable method for early detection of cardiovascular diseases with high accuracy and sensitivity.

### 3.3 Orthopedic Disease

Regarding the study of exosomes, the joint diseases that have received considerable attention are osteoarthritis (OA), osteonecrosis of the femoral head, and rheumatoid arthritis.^[^ ^189^ ^]^ Rheumatoid arthritis is a chronic inflammatory autoimmune disease primarily impacting the synovial joints, eventually leading to pain and disability in the affected individual. Arthritis affects approximately one out of every four individuals in the United States and is projected to affect 78.4 million people by 2040. However, the mortality rate associated with rheumatoid arthritis has significantly decreased over the past two decades, primarily due to efforts focused on early diagnosis, control, and treatment of the disease.^[^ ^190^, ^191^ ^]^ OA is another prevalent bone‐related disease that poses significant challenges in terms of treatment.^[^ ^189^ ^]^ Finding a reliable method for diagnosing bone diseases is crucial. One potential approach is to monitor exosomes and their contents as they have demonstrated promising diagnostic capabilities for various diseases.^[^ ^192^ ^]^ In this section, we emphasize studies conducted to elucidate the role of exosomes in the pathogenesis of joint diseases and their potential use as a source of biomarkers for diagnostic applications.

Exosome‐associated miRNAs have the potential to serve as valuable biomarkers for the diagnosis, monitoring, and progression of bone diseases.^[^ ^136^ ^]^ For example, one study has shown that the expression level of miR‐193b in plasma exosomes is reduced in patients with OA compared to healthy individuals, indicating its potential as a diagnostic marker.^[^ ^154^ ^]^ Exosomes and long non‐coding RNAs (lncRNAs) offer a potential alternative for diagnosing osteoarthritis in its early stages. LncRNAs are RNA molecules that play a role in cartilage development and the extracellular matrix degeneration of chondrocytes. A study investigated the expression of exosomal lncRNAs in OA patients’ plasma and synovial fluid to determine if they could serve as biomarkers for early‐stage detection.^[^ ^156^ ^]^ The findings revealed that the levels of exosomes in the synovial fluid of individuals with both early‐stage and late‐stage osteoarthritis were higher than control samples. This study supported the use of exosomes for detecting osteoarthritis and potentially aiding in the management of different stages of the disease. Importantly, the study noted that the levels of exosomal lncRNAs in the plasma of patients did not exhibit any significant difference compared to the control group. This discrepancy was attributed to the local nature of the disease, where changes in exosomal biomarkers are likely to be reflected at the site of damage rather than in the bloodstream.^[^ ^156^ ^]^

In support of the role of lncRNAs in joint diseases, a study conducted by Hongmei Xia et al. demonstrated the significant association between exosomes derived from various immune‐related cells (such as T cells, macrophages, dendritic cells, etc.) and the development and progression of bone‐related diseases. The cargo of these exosomes, particularly miRNAs and lncRNAs, have been found to play a crucial role in mediating inflammatory responses associated with bone diseases. This highlights the potential of exosomal miRNAs and lncRNAs as key regulators of inflammatory processes in bone‐related diseases.^[^ ^157^ ^]^

In contrast to their potential in diagnosing early‐stage osteoarthritis, exosomes may be used as a source of biomarkers to differentiate patients with early‐stage femoral head osteonecrosis from healthy individuals, however additional corroborating tests may be needed. In a study by Zhu et al., the serum level of exosomes in patients with steroid‐induced osteonecrosis of the femoral head was significantly decreased. The observed decrease in exosomal serum suggests that it could serve as a diagnostic factor for detection, however further research and larger‐scale studies are needed to evaluate the reliability and effectiveness of exosomal serum as a diagnostic tool for this condition.^[^ ^160^ ^]^

Exosomes released into the synovial fluid not only contribute to the progression of joint diseases but also can influence immune responses throughout the disease course. Their capacity to regulate immune responses makes exosomes a potential candidate for therapeutic applications in the treatment of joint diseases. The immunomodulatory properties of exosomes offer opportunities for developing exosome‐based therapies to target and modulate immune responses associated with joint diseases, as well as a mechanism to monitor treatment effectiveness.^[^ ^189^ ^]^

In a research study, it was confirmed that the level of lncRNA in exosomes, acting as macrophage activators, was elevated in patients with rheumatoid arthritis compared to those without the condition. However, there were no significant differences observed in the lncRNA levels in plasma exosomes between the two groups. This finding suggests that the specific role of lncRNAs in exosomes may be implicated in the immune response and pathogenesis of rheumatoid arthritis, emphasizing the importance of studying exosomal lncRNAs as potential biomarkers and therapeutic targets for this condition.^[^ ^156^, ^193^ ^]^

Exosomes derived from fibroblast‐like synoviocytes can influence macrophage migration. In particular, the presence of miR‐124 within these exosomes can inhibit the migration of macrophages. This indicates that exosomal miR‐124 plays a role in modulating the movement of macrophages, which is significant in the context of joint diseases and inflammation. Understanding the interplay between fibroblast‐like synoviocyte‐derived exosomes and macrophage migration, including the specific effects of miR‐124, provides valuable insights into the pathogenesis of various joint‐related conditions and their diagnosis.^[^ ^158^ ^]^

Exosomal miRNAs can also target macrophage autophagy, thereby influencing the progression of rheumatoid arthritis. These miRNAs, when delivered through exosomes, can modulate the autophagic process in macrophages. The dysregulation of macrophage autophagy is implicated in the pathogenesis of rheumatoid arthritis, and exosomal miRNAs can play a role in regulating this process. Understanding the mechanisms by which exosomal miRNAs impact macrophage autophagy can provide insights into potential therapeutic strategies for managing the progression of rheumatoid arthritis.^[^ ^159^, ^194^ ^]^

Exosomes, which carry immune response factors such as proinflammatory cytokines, can potentially contribute to bone structure damage. Fibroblast‐like synoviocytes in patients with rheumatoid arthritis release exosomes that contain elevated levels of TNF‐α.^[^ ^155^ ^]^ This, in turn, triggers the release of other inflammatory factors like IL‐1β and IL‐17, leading to joint damage. Additionally, platelet‐derived exosomes can also play a role in releasing inflammatory factors and promoting the progression of joint diseases. Furthermore, serum‐derived exosomes from rheumatoid arthritis patients contain HOX Transcription Intergenic RNA, which can contribute to bone and cartilage destruction. These findings underscore the involvement of exosomes in propagating inflammation and the destructive processes in joint diseases such as rheumatoid arthritis.^[^ ^155^ ^]^ In a recent systematic review by Schioppo et al., an analysis of ≈41 studies was conducted to establish statistical evidence regarding the relationship between exosome profiles and joint diseases.^[^ ^195^ ^]^ The review demonstrated an increase in the number of EVs, including exosomes, in patients with rheumatoid arthritis compared to healthy individuals. These findings highlight the potential of exosomes as diagnostic markers for joint diseases, providing statistical support for their role in disease diagnosis. The systematic review contributes valuable insights into the diagnostic application of exosomes in the field of joint diseases.^[^ ^195^ ^]^ In summary, there are various bone‐related diseases, and exosomes hold promise for distinguishing between different joint conditions. By studying the content of exosomes, such as specific molecules or biomarkers, it becomes possible to differentiate between different types of joint diseases. This potential offers a valuable avenue for improved diagnosis and understanding of these conditions.^[^ ^196^ ^]^

### 3.4 Neurological Diseases

Exosomes are released by various brain cells, e.g., dendritic, oligodendrocytes, microglia, neurons, and astrocytes in the central nervous system (CNS).^[^ ^197^, ^198^ ^]^ They play a significant role in intercellular communication, which means the modulation of cell‐to‐cell transmission of proteins and RNAs. Neuronal exosomes could serve various functions, such as synaptic plasticity, neural circuit development, and neuroprotection.^[^ ^199^ ^]^ They can be regulatory messengers of microglial function, as well as underlying inflammation‐induced synaptic alterations in the CNS.^[^ ^200^, ^201^ ^]^ The functions of exosomes could be neuroprotective or neurodegenerative.^[^ ^14^ ^]^ Neuroprotective function comprises the transfer of neuroprotective exosomal miRNAs into the recipient neural cell and removal of toxic proteins from them, while neurodegenerative roles involve the delivery of potentially toxic exosomal miRNAs, proteins, or lipids into the recipient neurons and spreading of toxic proteins in exosomes to the neighboring neural cells.^[^ ^14^, ^33^ ^]^ The accumulated toxic proteins in the cells, which are mutated or misfolded, cause oligomer formation and removal through the endosomal pathway, which either leads to lysosomal degradation or incorporation into MVBs and release into extracellular space as exosomes.^[^ ^14^, ^202^ ^]^

Accurate early diagnosis still demands further study, and the current biomarkers of NDs are not able to detect abnormal conditions in the initial or preclinical stage of the disease.^[^ ^114^ ^]^ The efficient biomarkers are necessary to detect and monitor NDs in early and progressive stages, respectively. The major sources of ND biomarkers are biofluids, comprising plasma, CSF, urine, serum and saliva. However, the evaluation of biomarkers derived from biofluids requires careful validation procedures and highly sensitive and specific biosensors. Moreover, the biofluid‐based hallmarks, e.g., brain‐derived biomarkers, typically have relatively low concentrations in the blood due to the blood‐brain barrier preventing free passage of molecules between the CNS and blood compartments^[^ ^9^, ^161^ ^]^ or could be invasive, e.g. in CSF‐derived sampling.^[^ ^199^ ^]^ Furthermore, the expression of the vast majority of biomarkers in non‐cerebral tissues, which are related to the pathological condition of NDs, may disrupt their measurement in the blood.^[^ ^9^ ^]^ In addition, the existence of heterophilic antibodies in the blood can lead to a reaction with the immunoassay antibodies, which may report falsely high or low results.^[^ ^161^ ^]^ Therefore, the novel biomarkers are essential to monitor the ND progression and enhance the early‐stage diagnostic sensitivity and specificity of four major ND disorders, including AD, PD, HD and ALS. Recent studies demonstrate that exosome‐based biomarkers have a great potential for accurate and specific diagnosis of various types of NDs and reflect the pathological factors in different stages. They can be isolated from multiple biofluids for clinical application as biomarkers. In this regard, it is worth mentioning that the development of novel specific and sensitive ND biosensors is significantly important in order to evaluate and detect exosomal biomarkers precisely. The suggested exosomal biomarkers for NDs have been summarized in Table 3.

#### 3.4.1 Alzheimer's Disease

The brain regulates the production and clearance of Aβ. Lysosomes are crucial in degrading excess proteins.^[^ ^33^ ^]^ While Aβ plaques are largely inert, the soluble Aβ oligomers around them are neurotoxic and correlate with AD severity.^[^ ^27^ ^]^ In both familial (early onset) and sporadic (late onset) AD, brain Aβ elimination is diminished.^[^ ^120^ ^]^ Exosomes play dual roles in this balance. Neuronal exosomes with normal Aβ and neuroprotective factors might act as cleansers for toxic Aβ, offering neuroprotection and aiding Aβ clearance.^[^ ^203^ ^]^ Exosomes from MSCs deliver active neprilysin, a key Aβ‐degrading enzyme in the brain^27^. Exosomes can also boost the degradation of soluble Aβ when taken up by microglia.^[^ ^204^ ^]^ Conversely, exosomes contribute to neuronal damage in AD. They are primary carriers that export soluble Aβ from cells, potentially seeding plaque formation.^[^ ^205^ ^]^ Exosomes may shift the balance between insoluble and soluble Aβ.^[^ ^27^ ^]^ Astrocyte‐derived exosomes have been shown to encourage soluble Aβ aggregation.^[^ ^206^ ^]^ Dysfunctional endosomal‐lysosomal systems in AD increase the release of toxic protein‐laden exosomes, promoting aggregation.^[^ ^207^ ^]^ Exosome‐released Aβ accumulation can suppress synaptic plasticity,^[^ ^33^ ^]^ disrupting neuronal signaling. Exosomes from APP‐expressing N2a cells have been observed to cluster around external Aβ, likely binding to their surface membranes.^[^ ^120^ ^]^ Exosome lipids convert extracellular insoluble Aβ to soluble forms, which are then taken up by microglia and released as a toxic Aβ variant in the extracellular fluid.^[^ ^208^ ^]^

The buildup of hyperphosphorylated p‐Tau into NFTs is a key AD characteristic.^[^ ^209^ ^]^ Unlike normal tau, p‐Tau cannot effectively stabilize microtubules.^[^ ^117^ ^]^ Phosphorylated p‐Tau linked with exosomes is found in the CSF of early AD patients, and its CSF levels align with cognitive decline.^[^ ^210^ ^]^ Notably, higher concentrations of abnormal p‐Tau in exosomes were seen in CSF from AD patients at advanced stages compared to early stages.^[^ ^33^ ^]^ The release of exosomes increases with neuronal activity. Exosomes, particularly from N2a cells, neurons, and microglia, are a primary way p‐Tau is secreted, emphasizing their role in AD pathology.^[^ ^211^, ^212^ ^]^ Still, a study showed that even when exosomal secretion was inhibited, and secreted p‐Tau reduced, there was little effect in blocking NLRP3 in microglia, pointing to non‐exosomal p‐Tau (soluble p‐Tau) as a potential trigger.^[^ ^209^ ^]^ Exosomes can also instigate neuroinflammation, heightening AD risk and neuron death. They spread inflammatory mediators in AD, like the cytokine TNF‐α and miR21, which can provoke AD inflammation and mitochondrial malfunction.^[^ ^213^ ^]^ Additionally, exosomes activate microglia, amplifying AD inflammation. Exosomes from astrocytes around senile plaques can induce other astrocytes’ apoptosis, pushing AD progression.^[^ ^2^ ^]^

Exosomal biomarkers have shown promise in the diagnosis and monitoring of AD. Exosomes carry miRNAs and proteins, which can be detected in biological fluids such as CSF, blood, saliva, and urine, making them easily accessible for diagnostic purposes.^[^ ^163^, ^164^, ^165^, ^214^, ^215^ ^]^ As mentioned, Aβ and Tau are two key proteins involved in the pathogenesis of AD. Both of these pathological features are hallmarks of AD. Exosomes have been found to contain both Aβ and tau, and their levels in exosomes have been associated with AD.^[^ ^216^ ^]^ For instance, a study found that exosomal tau with seeding activity is released from AD synapses, and its seeding potential is associated with Aβ.^[^ ^217^ ^]^ This suggests that exosomal tau may contribute to the spread of Tau pathology in AD, and its interaction with Aβ may play a role in this process. Another study found that there may be different subtypes of AD‐related protein aggregation, with some cases showing “amyloid‐first” and others showing “Tau‐first” patterns.^[^ ^162^ ^]^ In the “amyloid‐first” subtype, extensive neocortical Aβ precedes the aggregation of synaptic Tau. In contrast, in the “Tau‐first” subtype, Tau pathology may spread beyond the medial temporal lobe and interact with neocortical Aβ. This suggests that Aβ and Tau may begin as independent processes in spatially disconnected regions, with widespread neocortical Tau resulting from the local interaction of Aβ and Tau.^[^ ^162^ ^]^

Several studies have investigated the expression of exosomal miRNAs in AD patients and have identified specific miRNAs that are dysregulated in AD compared to healthy controls. These dysregulated miRNAs are involved in processes such as amyloid‐beta accumulation, tau‐dependent toxicity, inflammation, and neuronal death, which are all key features of AD pathology.^[^ ^163^, ^214^ ^]^ In CSF, exosomal miRNAs such as miR‐29c, miR‐136‐3p, miR‐16‐2, miR‐331‐5p, miR‐132‐5p, and miR‐485‐5p are significantly dysregulated in AD patients compared to healthy controls.^[^ ^214^ ^]^ In blood components such as serum and plasma, exosomal miRNAs such as miR‐342‐3p, miR‐141‐3p, miR‐342‐5p, miR‐23b‐3p, miR‐24‐3p, miR‐125b‐5p, and miR‐152‐3p are differentially expressed in AD patients compared to healthy controls.^[^ ^214^ ^]^ In addition to miRNAs, exosomal proteins have also been investigated as potential biomarkers for AD. A study focused on four synaptic proteins – growth associated protein 43 (GAP43), neurogranin, synaptosome associated protein 25 (SNAP25), and synaptotagmin 1‐ which have been implicated in AD pathology.^[^ ^164^ ^]^ The levels of these proteins in exosomes were significantly lower in the AD group compared to controls, and higher in the aMCI group compared to the AD group.^[^ ^164^ ^]^ Another study identified a panel of six proteins (A0A0G2JRQ6, C1QC, CO9, GP1BB, RSU1, and ADA10) in circulating plasma exosomes that were associated with AD.^[^ ^165^ ^]^

Despite the promising findings, several challenges need to be addressed. There is a lack of overlap between the different exosomal miRNAs found dysregulated across studies, which may be due to various factors such as the nature of the biological fluid, methods for exosome isolation, and miRNA quantification techniques.^[^ ^163^ ^]^ Furthermore, there is heterogeneity among the studies investigating exosomal proteins, and further research is needed to validate these biomarkers and establish standardized protocols for their detection.^[^ ^215^ ^]^

#### 3.4.2 Parkinson's Disease

PD is characterized by the loss of dopaminergic neurons in the substantia nigra pars compacta and the accumulation of Lewy bodies (LBs) in the brain, which are primarily composed of misfolded α‐syn.^[^ ^174^, ^218^ ^]^ This misfolded α‐syn triggers neuroinflammation, a hallmark of PD. Additional contributors to PD pathology include mutations in genes such as parkin, PTEN‐induced kinase, DJ‐1, and LRRK2.^[^ ^219^ ^]^ Environmental factors also exacerbate inflammation and neuronal loss. Brain immune cells like microglia and astrocytes release harmful substances, intensifying brain inflammation and neuronal injury. Recent evidence suggests that microglia‐induced immunoexcitotoxicity is a central player in PD,^[^ ^220^, ^221^ ^]^ with misfolded α‐syn being a primary activator of microglia.^[^ ^174^ ^]^ Certain microRNAs, including miR‐7 and miR‐137, have been implicated in PD progression by influencing related genes and promoting neuronal oxidative stress.^[^ ^222^ ^]^

Exosomes play a pivotal role in PD development.^[^ ^223^ ^]^ Derived from astrocytes and glial cells, they transfer misfolded proteins, notably toxic α‐syn, and inflammation‐linked miRNAs to neurons, fueling PD progression.^[^ ^223^ ^]^ Additionally, CNS‐derived exosomes stimulate α‐syn oligomerization in target cells, furthering PD.^[^ ^175^ ^]^ Notably, PD patients’ plasma and CNS‐derived exosomes show elevated α‐syn levels compared to controls.^[^ ^167^, ^224^ ^]^ Exosomes mediate intercellular communication, and with neurotoxic α‐syn, they spread toxicity to nearby cells. This results in α‐syn aggregation, microglial activation, and subsequent release of pro‐inflammatory cytokines like TNF‐α and IL‐1β. This cycle results in dopaminergic neuron death, exacerbating PD.^[^ ^222^ ^]^ Exosomes from activated microglia further boost neurodegeneration and PD progression.^[^ ^225^ ^]^

Pathological α‐syn transfers from neurons to astrocytes via exosomes, leading to inflammation in astrocytes.^[^ ^226^ ^]^ Exosomes also transport PD‐related miRNAs to cells, altering their genetic activity and accelerating neurodegeneration.^[^ ^222^ ^]^ For instance, serum exosomal miR‐137 levels were found elevated in PD mice, inducing neuronal oxidative stress.^[^ ^227^ ^]^ Differing exosomal miRNA levels were noted in the CSF of PD patients compared to healthy individuals.^[^ ^176^ ^]^ However, the role of exosomal miRNAs in PD remains under‐researched.^[^ ^121^ ^]^ While exosomes from neuronal cells clearly influence PD progression, further studies are needed to understand the transfer mechanisms of pathological factors through exosomes.

Currently, PD diagnosis relies on motor symptoms, which appear after a significant loss (≈70%) of dopaminergic neurons.^[^ ^228^ ^]^ There's a pressing need for non‐invasive blood biomarkers to detect early PD and distinguish it from disorders like MSA, PSP, and REM‐sleep behavior.^[^ ^169^, ^180^, ^229^ ^]^ Oligomeric α‐syn, pivotal in PD pathology, is a potential early detection biomarker. Detectable in blood and CSF, α‐syn concentrations could aid in PD diagnosis.^[^ ^230^ ^]^ However, challenges exist, including invasive CSF sampling, plasma sampling restrictions, and inconsistent results.^[^ ^32^ ^]^ Exosomes, present in minimally invasive biological fluids, could offer solutions for early PD diagnosis and monitoring.

Studies on α‐syn levels in CSF‐derived exosomes from PD patients have shown conflicting results. Guo et al. observed higher α‐syn levels in PD patients’ microglia‐derived exosomes compared to non‐PD counterparts.^[^ ^174^ ^]^ However, Stuendel et al. found reduced α‐syn in PD patients' CSF‐derived exosomes compared to healthy individuals.^[^ ^175^ ^]^ They believed that CSF exosomal α‐syn might not accurately reflect disease progression.^[^ ^175^ ^]^ Thus, while CNS‐derived exosomal α‐syn has potential as a PD biomarker, further research is essential for definitive conclusions.

Plasma is another potential biomarker source. Despite its easier extraction compared to CSF, directly confirming pathological α‐syn concentration in plasma is challenging due to the abundance of α‐syn from other blood cells. Hence, blood‐based exosomal α‐syn, like those from neurons and CNS, has been examined.^[^ ^166^, ^167^ ^]^ Shi et al. found that plasma‐derived CNS exosomal α‐syn levels were notably higher in PD patients and correlated with disease severity.^[^ ^167^ ^]^ Additionally, they suggested that plasma exosomal α‐syn might be a reliable PD biomarker.^[^ ^167^ ^]^ Their research also revealed elevated Tau levels in PD patients' CNS‐derived plasma exosomes, correlating with CSF total Tau and p‐Tau.^[^ ^178^ ^]^ Notably, significant differences were identified in the ratios of oligomeric to total α‐syn, and oligomeric phosphorylated α‐syn to total phosphorylated α‐syn, in the plasma exosomes of PD patients compared to healthy controls, indicating potential early‐stage PD diagnosis markers.^[^ ^32^ ^]^ Elevated oligomeric α‐syn/total α‐syn ratios were also identified in PD patients' saliva exosomes.^[^ ^188^ ^]^

Urine could be utilized as a noninvasive source of exosomal PD biomarkers. LRRK2 and DJ‐1 proteins in urine exosomes might serve as a PD biomarker. One study reported that the phosphorylated Ser‐1292 LRRK2, which is the result of the specific G2019S mutation, to total LRRK2 ratio in urine exosomes was increased in G2019S mutant carriers with PD compared with non‐mutant idiopathic PD patients and non‐PD.^[^ ^187^ ^]^ They suggested that this ratio might be used to predict the risk of PD symptoms development among LRRK2 G2019S mutation carriers.^[^ ^187^ ^]^ In another study conducted by this group, they concluded that the levels of Ser(P)‐1292 LRRK2 were increased in idiopathic PD patients compared with healthy controls and higher in men than in women.^[^ ^186^ ^]^ Contrary to these findings, Ho et al. evaluated the levels of LRRK2, α‐syn, and DJ‐1 in urine exosomes in PD patients, resulting in no difference in the LRRK2 protein between PD and non‐PD groups. Based on their outcomes, the level of DJ‐1 protein in urine exosomes was significantly higher in male PD patients and strongly dependent on gender.^[^ ^185^ ^]^ They could not detect *α*‐syn in urine samples, suggesting that DJ‐1 protein from urine exosomes could be used to diagnose PD in males.^[^ ^185^ ^]^ However, another research group analyzed the level of DJ‐1 in plasma and plasma neural‐derived exosomes in PD patients versus non‐PD and reported that although the level of neural‐derived exosomal DJ‐1 increased significantly in PD patients, there were no significant differences in DJ‐1 level of plasma among PD and non‐PD.^[^ ^166^ ^]^ Based on these results, further studies on DJ‐1 and LRRK2 are essential to provide supportive evidence and prove their accuracy to be considered reliable PD biomarkers for early detection and progression.

Serum exosomal α‐syn has also been evaluated as a potential PD indicator. Si et al. reported decreased serum CNS‐exosome α‐syn levels in PD patients compared to non‐PD individuals.^[^ ^179^ ^]^ Contrarily, Dutta et al. studied α‐syn levels in neuronal and oligodendroglial exosomes from serum, aiming to differentiate PD from MSA.^[^ ^180^ ^]^ They observed significant α‐syn elevations in both MSA and PD patients compared to healthy controls, with higher levels in MSA.^[^ ^180^ ^]^ Their findings suggest that measuring exosomal α‐syn might help distinguish MSA from PD.

Besides α‐syn, other exosomal biomarkers in plasma have been proposed for PD. Three exosomal proteins, namely clusterin, complement C1r subcomponent, and apolipoprotein A1, were examined across PD stages and proposed as potential indicators and trackers of PD progression.^[^ ^168^ ^]^ A study compared exosome levels from neurons, astrocytes, and oligodendrocytes in the plasma of PD, MSA, PSP, and healthy participants.^[^ ^169^ ^]^ They found neuron‐derived exosomes significantly elevated in PD patients compared to healthy controls and MSA. While all brain‐derived exosome levels were higher in advanced PD than in mild cases or non‐PD individuals, only neuron‐derived and oligodendrocyte‐derived exosomes were notably elevated in early PD versus non‐PD groups. Consequently, neuron‐derived exosomes might be suitable for early PD detection, and oligodendrocyte‐derived exosomes might track PD progression.^[^ ^169^ ^]^ Additionally, blood‐based exosomal acetylcholinesterase (AChE) activity, which was found to decrease in PD patients compared to healthy individuals, could serve as a reliable early PD diagnostic and prognostic marker.^[^ ^170^ ^]^

Exosomes also contain PD‐related miRNAs, which hold promise for early PD diagnosis. Studies have shown that exosomal miRNAs from CSF, plasma, and serum of PD patients might act as PD indicators.^[^ ^177^, ^181^, ^182^ ^]^ The potential exosomal miRNA biomarkers for PD are detailed in Table 3. While these exosomal miRNAs offer non‐ or minimally invasive sampling and early detection, their broader adoption requires extensive studies, database development, and enhanced sampling and data analysis methods for further validation and discovery of new PD‐diagnosing miRNAs.^[^ ^169^, ^224^ ^]^

## 4 Perspectives on the Identification of Extracellular Vesicles

The bioavailability and diversity of exosomes in bodily fluids, such as saliva, plasma, and urine, make them an ideal candidate for disease detection through liquid biopsies. Additionally, exosomes contain many biologically relevant constituents such as proteins, nucleic acids, and lipids.^[^ ^231^ ^]^ This is clinically relevant since rapid disease detection can become accessible during medical physical examinations. Research efforts to identify novel biomarkers, understand disease‐related pathways, and develop novel detection devices have increased over the last five years. Recent advances in exosome isolation and detection focus on optimizing parameters such as limit of detection (LOD), smaller sample volume, sample manipulation, cost‐effectiveness, standardization and quality control, clinical translatability, selectivity, efficiency, stability, and scalability.^[^ ^231^, ^232^, ^233^, ^234^, ^235^, ^236^, ^237^, ^238^ ^]^

Zhang et al. eliminated the use of antibodies and developed a label‐free sensor for exosome detection.^[^ ^239^ ^]^ They used non‐small cell lung cancer (NSCLC) as a model cell line to demonstrate the efficiency of their novel Antibody and label‐free molecularly imprinted polymer (MIP)‐based impediment sensor. This sensor functions by measuring charge conductance due to selective target binding. Using a glassy carbon electrode (GCE), cholesterol molecules, and electrochemical polymerization, the sensor was able to recognize and bind to NSCLC cell‐derived exosomes without the need for antibodies. The MIP sensor detects exosomes using changes in electrical impedance when exosomes bind to the polymer, leading to detection with increased sensitivity and selectivity.^[^ ^239^ ^]^ This method addresses the optimization parameters of LOD and cost‐effectiveness. A reduction of LOD to the range of pM/mL was observed, which demonstrates an overall increase in detection sensitivity. Additionally, eliminating the use of antibodies for detection reduces cost and makes this method cost‐effective since antibodies add additional cost to the overall experiment. Finally, this approach also eliminates batch variability introduced by using antibodies since antibodies can vary during each production cycle, therefore making the process standard and reproducible.^[^ ^239^ ^]^

Similarly, Li et al. developed a novel label‐free exosome detection method using a functionalized carbon nanotube field‐effect transistor (CNT‐FET) biosensor.^[^ ^232^ ^]^ CNT‐FET is a highly selective exosomal protein detection method that allows for real‐time monitoring using CNT electrical properties to transduce binding events between exosomal proteins and the functionalized surface.^[^ ^232^ ^]^ Both Zhang et al. and Li et al. demonstrate the potential of their label‐free method as early diagnostic and monitoring tools due to lower LOD and enhanced selectivity.^[^ ^231^, ^232^ ^]^ Not only is there a need for novel isolation and detection‐based optimizations, but there is also the need for mechanism‐based disease understanding since cancer, neurological diseases, and cardiovascular diseases are multifactorial.

Shou et al. investigated the role of exosome‐derived miR‐154‐5p in the progression and angiogenesis of esophageal squamous cell carcinoma (ESCC).^[^ ^240^ ^]^ Exosomes for normal esophageal epithelial cells contain miR‐154‐5p, which is downregulated during ESCC. Researchers claim that miR‐154‐5p attenuates ESCC progression by inhibiting cell proliferation, migration, and invasion. miR‐154‐5p promotes an anti‐angiogenic effect by targeting kinesin family member 14 (KIF 14), a protein involved in angiogenesis. The project highlights the use of exosomal miRNA as a therapeutic biomarker for suppressing tumor growth and angiogenesis through inhibition of KIF‐14.^[^ ^240^ ^]^

Exosomes can serve as both drug‐delivery vesicles and drug resistance vesicles because they can carry different biomolecules, including disease progressive biomolecules and therapeutic biomolecules. In this regard, radiation‐induced exosomes and their role in tumor progression and radioresistance in hepatocellular carcinoma (HCC) have been explored.^[^ ^241^ ^]^ Radiation is a common treatment for HCC, but over time, radiotherapy can induce the release of exosomes from HCC cells, which leads to tumor progression and radio resistance. Radiation‐induced exosomes lead to HCC progression by facilitating angiogenesis, immune invasion, and epithelial‐mesenchymal transition (EMT). Researchers can also enhance radioresistance by transferring specific molecules, such as miRNAs and proteins, to recipient cells, promoting DNA repair and cell survival. Further analysis of exosome‐based cellular communication mechanisms can provide an understanding of how radio resistance works and strategies to overcome it for improving the effectiveness of radiation therapy for HCC proteins.^[^ ^241^ ^]^

Advances in existing immuno‐capture‐based exosome isolation methods are also being made. Jang et al. used magnetic transferrin nanoparticles (MTNs) to isolate neurological disease‐derived biomarkers.^[^ ^236^ ^]^ MTNs are functionalized nanoparticles that bind to transferrin receptors on exosome surfaces and allow for exosome isolation from complex samples. Identification and analysis of biomarkers from neurological disease‐derived exosomes can contribute to early diagnosis and monitoring of neurological diseases by using MTNs to combine the advantages of magnetic separation and specific immuno‐targeting capabilities. overall, this study facilitates the study and development of exosome‐based diagnostics and therapeutics in NDs. Ultimately, the potential of MTN‐based isolation and detection can broaden to other disease models.^[^ ^236^ ^]^

Conventionally, tools such as ultracentrifugation, density gradient separation, precipitation, and size exclusion chromatography have been used for exosome isolation, followed by nucleic acid amplification techniques (quantitative PCR), microarrays, fluorescence‐based assays, enzyme‐linked immunosorbent assays (ELISAs), and next‐gen sequencing for detecting specific disease‐related exosomal miRNAs.^[^ ^231^, ^242^ ^]^ This enables the detection and characterization of genetic alterations and molecular profiles associated with various diseases.^[^ ^235^ ^]^ miRNAs play a crucial role in cellular communication. Techniques such as nanoparticle tracking analysis, flow cytometry, electron microscopy, mass spectrometry, and RNA sequencing are also used to determine size, morphology, and additional biomarkers.^[^ ^234^ ^]^ Currently, there is an increased use of microfluidics to develop a convergent platform for immunocapture‐based isolation, characterization, and analysis of exosomal content.^[^ ^234^ ^]^ As discussed by Pishbin et al., using AD as a disease model, miRNA profiling can improve understanding and disease management for AD while microfluidics can enhance exosome enrichment and isolation, leading to efficient capture and profiling of various exosomal biomarkers.^[^ ^242^ ^]^ Microfluidics can provide a sterile and streamlined environment for exosomal research while also enhancing efficiency, scalability, and sensitivity.^[^ ^234^ ^]^

Advances in microfluidic approaches are shown using paper/poly (methyl methacrylate) (PMMA) hybrid devices as a novel microvalve controlled device that focuses on the isolation and analysis of exosomes.^[^ ^233^ ^]^ Hu et al. used this device for efficient exosome isolation using precise fluid flow control and simple manipulation.^[^ ^233^ ^]^ Researchers claim that this method provides a reliable platform for exosome analysis by increasing capture efficiency and sample purity. The device also permits integration of sample loading, washing, and elution streamlining and automating exosome isolation and analysis. This method is cost‐effective and user‐friendly compared to individual experiments requiring more materials and complicated protocols. Finally, this allows for further exosomal content, such as miRNA, analysis using pure exosomes.^[^ ^233^ ^]^

Instead of miRNAs, Wang et al., highlight comparing differential expression of circRNAs in tumor patients and healthy patients to demonstrate circRNAs potential as biomarkers and therapeutic targets for gastrointestinal tumors.^[^ ^243^ ^]^ Researchers claim that circRNAs can serve as potential prognostic markers for predicting tumor progression and outcome since they can regulate gene expression and signal pathways in recipient cells and hence are attractive for novel therapeutic interventions for gastrointestinal tumors. Upon further analysis, differential expression profiles of circRNAs can also be used to detect and monitor other cancers.^[^ ^243^ ^]^

Different from individual analyte detection, Rani et al. used high‐resolution liquid chromatography‐mass spectrometry (LC‐MS) to identify and quantify multiple proteins in salivary exosomes to evaluate their role in the progression of cognitive impairment in AD.^[^ ^244^ ^]^ Differential protein expression patterns in salivary exosomes between individuals with cognitive impairment and those with AD have potential as biomarkers for disease progression. This can also allow for early diagnosis and intervention strategies.^[^ ^244^ ^]^

In addition to disease detection, the regenerative potential of exosomes is also valuable as it can aid in post‐treatment modification. Codispoti et al. describe the role of exosomes in regenerative medicine through the novel approach of NANOmetric BIO‐Banked MSC‐Derived exosome (NANOBIOME).^[^ ^237^ ^]^ Exosomes derived from MSCs have regenerative and immunomodulatory properties which make them a potential candidate for various therapeutic applications. The advantages of NANOBIOME include stability, scalability, and potential off‐the‐shelf use compared to traditional cell‐based therapies.^[^ ^237^ ^]^

Current research highlights the need for standardization and quality control measures in exosome analysis to translate the use of exosomes for clinical applications.^[^ ^234^ ^]^ The future of liquid biopsies and liquid detection research is moving toward integrating multiple analytes for disease analysis, developing more sensitive and specific assays, label‐free detection, lower LOD, and smaller sample volume.^[^ ^235^ ^]^ Researchers establish the importance of standardizing and validating liquid biopsy assays for reliable and reproducible clinical use.

## 5 Conclusions and Perspectives

The diagnosis and therapy of chronic diseases, such as cancer, cardiovascular disease, neurodegenerative disease, and orthopedic disease, hold special significance in the field of healthcare when compared to other chronic conditions. Several factors contribute to their elevated importance. First and foremost, these four chronic diseases collectively substantially impact global public health due to their high prevalence and the severe consequences they bring. They affect millions worldwide and impose a significant societal and economic burden. Moreover, cancer, cardiovascular disease, neurodegenerative diseases like Alzheimer's and Parkinson's, and orthopedic diseases such as osteoarthritis are characterized by their complexity and severity. They often lead to debilitating symptoms, reduced quality of life, and long‐term disability. Early diagnosis and appropriate therapy are paramount because they can profoundly influence the course of these diseases. For example, detecting cancer early can lead to more effective treatments and improved survival rates. Similarly, timely interventions for cardiovascular disease can prevent heart attacks and strokes.

Exosomes serve as essential mediators of intercellular communication, playing critical roles in various physiological and pathological processes. Their diagnostic potential is evident in diverse chronic diseases, including cancer, cardiovascular diseases, orthopedic conditions, and neurodegenerative disorders. Notably, they carry a cargo of proteins, nucleic acids, and lipids, making them valuable diagnostic biomarkers. Beyond diagnostics, exosomes show promise in therapeutics, serving as effective drug delivery systems and agents in regenerative medicine. They possess the capability to transport therapeutic cargoes and modulate cellular functions, leading to innovative pharmacological approaches for diagnostics and therapy. Exosomes offer several advantages over synthetic nanomaterials, such as drug delivery systems and diagnostic tools, biocompatibility, reduced toxicity, stability and protection of cargo, and the ability to cross biological barriers.

Exosomes have emerged as a promising liquid biopsy tool, offering a non‐invasive method for disease diagnosis and monitoring. Their use enables real‐time tracking of treatment response and disease progression, presenting numerous advantages for various applications. One of the notable advantages of exosomes is their cost‐effectiveness and user‐friendly nature, making them highly accessible for research, diagnosis, therapeutics, and biomarker discovery.^[^ ^233^, ^235^ ^]^ This cost efficiency opens up new possibilities for widespread implementation and further advances in the field. In the realm of cancer detection and monitoring, exosomes have proven to be instrumental in the analysis of circulating tumor cells (CTCs), circulating tumor DNA (ctDNA), and cell‐free RNA. This liquid biopsy approach provides valuable insights into cancer progression, aiding in early detection and assessment of treatment efficacy.^[^ ^235^ ^]^

Further research on exosomes and their functional mechanisms is essential, as it will significantly enhance our understanding of disease pathogenesis, ultimately contributing to the development of groundbreaking pharmacological interventions.^[^ ^245^ ^]^ Exosomes' unique advantage in personalized medicine lies in their ability to contain biomarkers that assess interindividual variability in drug exposure and metabolism. This opens the door to tailored treatments, where drug regimens can be optimized for individual patients, improving treatment outcomes and minimizing adverse effects.

However, several challenges need to be addressed to realize their widespread clinical application, alongside exploring future perspectives to maximize their benefits. One of the primary challenges is the standardization of isolation and characterization methods. Currently, various techniques are employed, leading to inconsistent results. Developing standardized methods will ensure reproducibility and comparability across different studies, enhancing the reliability of exosome‐based diagnostics and therapies. Scalability and production efficiency are also critical issues. As the demand for exosome‐based therapies grows, scalable and efficient production methods become essential. Current isolation methods can be time‐consuming and yield limited quantities of exosomes. Improving production efficiency and cost‐effectiveness will be critical for large‐scale clinical applications. Another crucial challenge is the ability to target specific cell types effectively. For therapeutic applications, efficient targeting of specific cell types or tissues is vital to minimize off‐target effects and maximize therapeutic efficacy. Engineering exosomes to express specific targeting ligands or receptors will enhance their ability to reach the intended sites of action. Ensuring the stability of exosomes during storage and transportation is also a concern. Exosomes are sensitive to various environmental conditions, such as temperature and pH. Developing storage strategies that maintain exosome integrity will be essential for practical applications. Before exosomes can be widely used in patients, their safety profile needs to be thoroughly evaluated. Concerns regarding potential immunogenicity and off‐target effects must be addressed through comprehensive preclinical studies and clinical trials.

In the future, exosomes hold tremendous promise for personalized medicine. By utilizing the cargo of exosomes as diagnostic biomarkers, clinicians can tailor treatments to individual patients, improving treatment outcomes and reducing adverse effects. Additionally, engineered exosomes can serve as highly efficient drug delivery systems. Future research may focus on optimizing exosome cargo loading to precisely target disease‐specific molecules, opening new avenues for targeted therapies. Combining exosome‐based therapies with conventional treatments or other novel therapeutic approaches could result in synergistic effects and enhanced treatment efficacy, which warrants further investigation. Exosomes also offer a minimally invasive approach for liquid biopsies, providing valuable diagnostic information without the need for invasive tissue sampling. Advancements in exosome‐based liquid biopsies may revolutionize cancer diagnostics and monitoring. Furthermore, exosomes show immense potential in crossing the BBB, making them attractive candidates for diagnosing and treating neurological diseases. Future research may focus on harnessing this capability for targeted neurological therapies.

## Conflict of Interest

The authors declare no conflict of interest.

## Author Contributions

F.E. performed conceptualization, wrote the original draft, reviewed, edited, and photo design; A.K. wrote Section 4, and part of Section 5, reviewed, edited and photo design; S.G. wrote Sections 2.1 and 3.3, and photo design; S.A. wrote Sections 3.1 and 3.2. reviewed, edited and photo design; K.D. performed conceptualization, funding acquisition; supervision, reviewed and edited.
